# Hidden genomic features of an invasive malaria vector, *Anopheles stephensi*, revealed by a chromosome-level genome assembly

**DOI:** 10.1186/s12915-021-00963-z

**Published:** 2021-02-10

**Authors:** Mahul Chakraborty, Arunachalam Ramaiah, Adriana Adolfi, Paige Halas, Bhagyashree Kaduskar, Luna Thanh Ngo, Suvratha Jayaprasad, Kiran Paul, Saurabh Whadgar, Subhashini Srinivasan, Suresh Subramani, Ethan Bier, Anthony A. James, J. J. Emerson

**Affiliations:** 1 Department of Ecology and Evolutionary Biology, University of California, Irvine, CA 92697 USA; 2 Section of Cell and Developmental Biology, University of California, San Diego, La Jolla, CA 92093-0335 USA; 3 Tata Institute for Genetics and Society, Center at inStem, Bangalore, Karnataka 560065 India; 4 Department of Microbiology & Molecular Genetics, University of California, Irvine, CA 92697 USA; 5 Institute of Bioinformatics and Applied Biotechnology, Bangalore, KA 560100 India; 6 Section of Molecular Biology, University of California, San Diego, La Jolla, CA 92093-0322 USA; 7 Tata Institute for Genetics and Society, University of California, San Diego, La Jolla, CA 92093-0335 USA; 8 Department of Molecular Biology & Biochemistry, University of California, Irvine, CA 92697 USA; 9 Center for Complex Biological Systems, University of California, Irvine, CA 92697 USA

**Keywords:** *Anopheles stephensi*, Reference-quality assembly, Transposable elements, Insecticide resistance, Y chromosome, Malaria control, Blood feeding, Structural variation

## Abstract

**Background:**

The mosquito *Anopheles stephensi* is a vector of urban malaria in Asia that recently invaded Africa. Studying the genetic basis of vectorial capacity and engineering genetic interventions are both impeded by limitations of a vector’s genome assembly. The existing assemblies of *An. stephensi* are draft-quality and contain thousands of sequence gaps, potentially missing genetic elements important for its biology and evolution.

**Results:**

To access previously intractable genomic regions, we generated a reference-grade genome assembly and full transcript annotations that achieve a new standard for reference genomes of disease vectors. Here, we report novel species-specific transposable element (TE) families and insertions in functional genetic elements, demonstrating the widespread role of TEs in genome evolution and phenotypic variation. We discovered 29 previously hidden members of insecticide resistance genes, uncovering new candidate genetic elements for the widespread insecticide resistance observed in *An. stephensi*. We identified 2.4 Mb of the Y chromosome and seven new male-linked gene candidates, representing the most extensive coverage of the Y chromosome in any mosquito. By tracking full-length mRNA for > 15 days following blood feeding, we discover distinct roles of previously uncharacterized genes in blood metabolism and female reproduction. The Y-linked heterochromatin landscape reveals extensive accumulation of long-terminal repeat retrotransposons throughout the evolution and degeneration of this chromosome. Finally, we identify a novel Y-linked putative transcription factor that is expressed constitutively throughout male development and adulthood, suggesting an important role.

**Conclusion:**

Collectively, these results and resources underscore the significance of previously hidden genomic elements in the biology of malaria mosquitoes and will accelerate the development of genetic control strategies of malaria transmission.

**Supplementary Information:**

The online version contains supplementary material available at 10.1186/s12915-021-00963-z.

## Background

Mosquitoes transmit the largest number of arthropod vector-borne diseases (i.e., malaria, dengue, Zika, yellow fever, and Chikungunya) in humans and animals globally [1, 2]. The complex disease human malaria is caused by *Plasmodium* parasites, which are transmitted by female *Anopheles* mosquitoes [3]. Nearly 20 years ago, sequencing of the *An. gambiae* genome catalyzed rapid growth in genetics and genomics research in this important vector of sub-Saharan Africa [4–6]. However, the lack of comparable genomic resources in other malaria vectors has impeded progress in understanding and control of the spread of this deadly disease in other continents [7, 8].

*Anopheles stephensi* is the primary vector of urban malaria in the Indian subcontinent and the Middle East and an emerging malaria vector in Africa [9, 10]. The species is so invasive that without immediate control, it is predicted to become a major urban malaria vector in Africa, putting 126 million urban Africans at risk [11]. Genetic strategies (e.g., clustered regularly interspaced short palindromic repeats (CRISPR) gene drives) that suppress or modify vector populations are powerful means to curb malaria transmission [12, 13]. The success of these strategies depends on the availability of accurate and complete genomic target sequences and variants segregating within them [12, 14]. However, functionally important genetic elements and variants within them often consist of repetitive sequences that are either mis-assembled or completely missed in draft-quality genome assemblies [15, 16]. Despite being a pioneering model for transgenics and CRISPR gene drive in malaria vectors [13, 17], the community studying *An. stephensi* still relies on draft genome assemblies that do not achieve the completeness and contiguity of reference-grade genomes to reveal all the hidden genetic features [7, 18, 19]. In particular, this limitation obscures genes and repetitive genetic elements that are potentially relevant for understanding parasite transmission or for managing vector populations [20]. We therefore generated a high-quality reference genome for a laboratory strain UCISS2018 (see the “Materials and methods” section) of this mosquito sampled from the Indian subcontinent (Additional file 1: Figure S1) using deep coverage long reads plus Hi-C scaffolding and then annotated it by full-length mRNA sequencing (Iso-Seq). These resources facilitate characterization of regions of the genome less accessible to previous efforts, including gene families associated with insecticide resistance, targets for gene-drive interventions, and recalcitrant regions of the genome rich in repeats, including the Y chromosome.

## Results

### A reference-grade assembly of *An. stephensi*

**Fig. 1:**
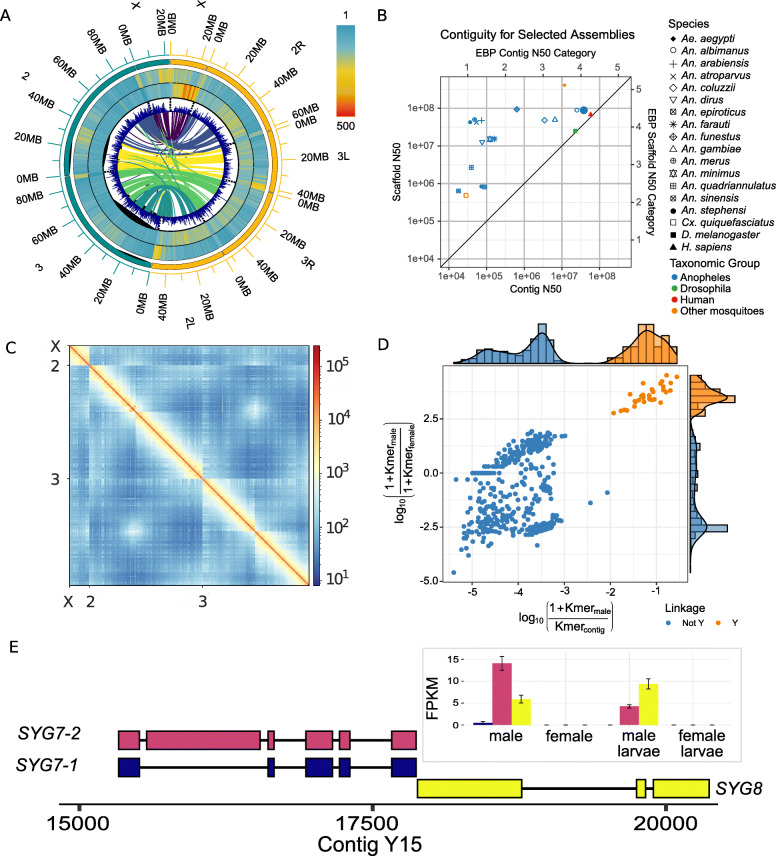
*Anopheles stephensi* genome assembly. **a** Distribution of repeats, gene content, and synteny between *An. stephensi* (left, green) and *An. gambiae* (right, yellow) genomes. Each successive track from outside to inside represents TE density, satellite density (Additional file 1: Figures S2 and S3), and gene density across the chromosome arms in 500-kb windows. The innermost track describes the syntenic relationship between *An. stephensi* and *An. gambiae* chromosome arms. **b** Contiguities of published genome assemblies of *Anopheles* malaria vectors, *Culex*, *Aedes aegypti*, human (GRCh38.p13), and the model organism *D. melanogaster*. Among the mosquito vectors, *An. stephensi* assembly reported in the current study is the only genome that matches the Earth BioGenome Project (EBP) standard of the human and the *Drosophila* genomes [21]. **c** Hi-C contact map of the *An. stephensi* scaffolds. The density of Hi-C contacts is highest at the diagonals, suggesting consistency between assembly and the Hi-C map. **d** Identification of putative Y contigs using the density of male-specific k-mers on the *x*-axis and the ratio of male and female k-mers on the *y*-axis. **e** Transcripts of *SYG7* and *SYG8*, two new Y-linked genes as revealed by the uniquely mapping Iso-seq reads. SYG7 has two isoforms. Inset: transcript abundance of *SYG7* and *SYG8* in male and female adults and larvae. As shown here, neither gene is expressed in females

*Anopheles stephensi* has three major gene-rich chromosomes (X, 2, 3) and a gene-poor, heterochromatic Y chromosome (Fig. 1a) [18]. The size of the published most contiguous draft assembly of the *An. stephensi* genome was 221 Mb and had 23,371 scaffolds with N50 of 1.59 Mb, meaning that half of the assembly is found in scaffolds < 1.59 Mb (Additional file 1: Table S1) [18]. The longest scaffold of this assembly was 5.9 Mb and 11.8 Mb of gaps in the assembly was filled with Ns. In the new reference assembly, the major chromosomes are represented by just three sequences (scaffold N50 = 88.7 Mb; contig N50 = 38 Mb; N50 = 50% of the genome is contained within sequence of this length or longer), making this assembly comparable to the *Drosophila melanogaster* reference assembly, widely considered a gold standard for metazoan genome assembly [22] (Fig. 1b, Table 1, Additional file 1: Figures S4 and S5, Additional file 1: Table S1). The new reference assembly has 89% (205/235 Mb) of the estimated *An. stephensi* physical haploid genome assigned to chromosomes, which parallels the assembly completeness of *An. gambiae* where 88% (230/265 Mb of physical genome size) of the assembled genome is placed into chromosomes (see the “Materials and methods” section) (Fig. 1).

**Table 1:** Summary of genome assembly and annotation statistics

Genomic features	Value
Total length (bp)	250,632,892
Contig number	566
Contig N50 (bp)	38,117,870
Scaffold number	560^
Scaffold N50 (bp)	88,747,609*
L50	2
GC content (%)	44.91%
Maximum scaffold length	93,706,523
Minimum scaffold length	1727
*N*’s per 100 kb	3.60
Alternative haplotypes, bp (# scaffolds)	15,743,318 (169)
Unclassified contigs, bp (# scaffolds)	20,118,109 (355)
Putative Y chromosome, bp (# scaffolds)	2,431,719 (33)
3 major chromosomes X, 2, and 3, bp (# scaffolds)	205,167,748 (3)
Predicted genes	14,966
Predicted transcripts	16,559
5′ UTR	9791
3′ UTR	9290
tRNAs	503

^Except three major chromosomes, we kept others as contigs; *Scaffold N50 is the length of chr3

The new *An. stephensi* assembly recovers 99.2% of 3285 complete single copy Diptera orthologs (i.e., Benchmarking Universal Single Copy Orthologs or BUSCOs) [23]. The reference assembly of the *D. melanogaster* genome captures 99.1% of BUSCOs, indicating that their completeness is comparable (Table 1, Additional file 1: Figure S5, Additional file 1: supplementary text). The new *An. stephensi* assembly not only achieves significant improvements over the existing draft assembly (1044-fold and 56-fold increase in contig N50 and scaffold N50, respectively, and a 97% reduction in assembly gaps), but it is also more contiguous and complete than the latest versions of published assemblies of *Anopheles* species (single copy BUSCOs 93.7–98.9%) [7, 18, 19], including the extensively studied African malaria vector, *An. gambiae* (AgamP4) genome (BUSCO 97.4%, contig N50 = 85 kb, scaffold N50 = 49 Mb), arguably the best characterized genome among malaria vectors (Fig. 1a, Table 1, Additional file 1: Table S1, Additional file 1: Figures S3 and S5) [7, 20, 24–27]. Furthermore, the concordance of short reads mapped to the assembly suggests that assembly errors are rare (short read consensus quality value or QV = 49.2, or ~ 1 discrepancy per 83 kb), which is further supported by uniformly mapping long reads and a high-resolution Hi-C contact map (Fig. 1c, Table 1, Additional file 1: Figure S1). Among the mosquito vectors, the *An. stephensi* assembly reported here is the genome that matches most closely the standards achieved by the human and the *Drosophila* genomes (Fig. 1b) and is the only *Anopheles* genome to meet the standards of the Earth BioGenome Project (EBP) [21] for both contiguity and accuracy.

Furthermore, assessment of annotation showed that 92% of genes are annotated with < 0.5 annotation edit distance (AED), which surpasses the recommended score (> 90%) for gold standard annotations (Campbell et al.) [115]. The Pfam content score (65.4%) is also above the recommended range 55–65%, indicating that the *An. stephensi* proteome is well annotated. Comparison of gene model annotations of our assembly with the draft assembly showed that 24.4% of 14,966 genes were unique in our assembly. An additional 1429 genes in our assembly were split over > 1 contig in the older most contiguous *An. stephensi* assembly [18]. Collectively, such evidence suggests that our assembly recovered previously unassembled functional genome sequences. We also assembled 33 putative Y contigs totaling 2.4 Mb, representing the most extensive Y chromosome sequence yet recovered in any *Anopheles* species [28] (Fig. 1d to f) [7, 18, 28]. Finally, to assist disease interventions using endosymbionts [29], we assembled de novo the first complete genome of the facultative endosymbiont *Serratia marcescens* from *Anopheles* using sequences identified in the *An. stephensi* long read data (Additional file 1: Figure S4).

### Transposable elements

**Fig. 2:**
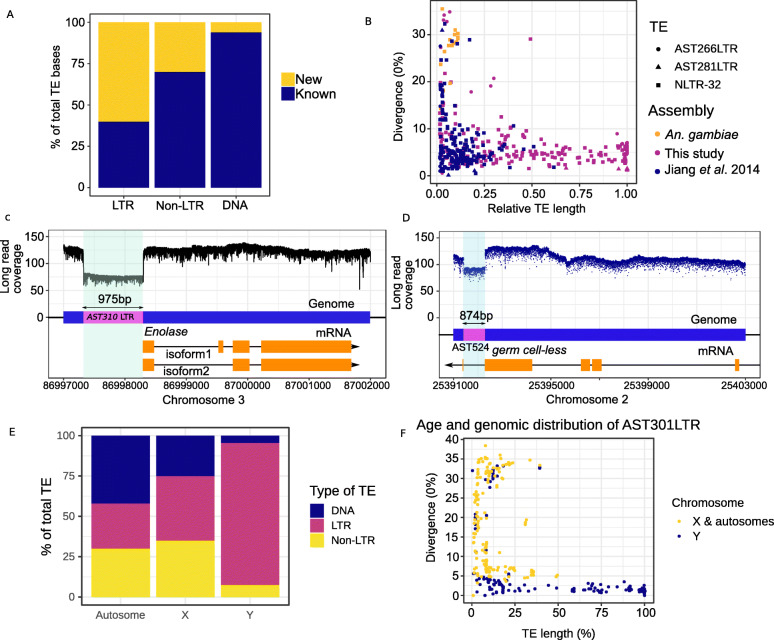
Transposable elements (TEs) and their role in genetic variation in *An. stephensi.* **a** Proportion of TEs (counted in bp) that were uncovered by the new reference assembly of *An. stephensi*. Many LTR and non-LTR TEs are identified for the first time. **b** Similar to many other LTR and non-LTR retrotranspososons, LTR elements AST266LTR and AST281LTR and non-LTR element NLTR-32 do not have any closely related counterpart in *An. gambiae*. As shown here, only small parts of these TE sequences were known. **c** Insertion of a polymorphic LTR fragment immediately upstream of the highly conserved gene *Enolase*. The insertion creates a gap between the promoters and the transcription start sites in half of the alleles and may disrupt transcription of the gene. **d** A polymorphic DNA element AST524 located in the 3′ UTR of *gcl* creates a null *gcl* allele. **e** Comparison of TE compositions in the autosomal, X, and Y chromosomal sequences. Not only are most of the Y sequences repeats (Additional file 1: Figure S6), but the majority of Y TEs are LTR elements. **f** LTR retrotransposon AST301 is present in intact copies only in Y contigs. Its counterparts in the autosomes and X chromosome are fragmented and more diverged than the AST301 sequences found on the Y sequences

As naturally occurring driving genetic elements, transposable elements (TEs) are invaluable for synthetic drives [30, 31] and transgenic tools [17, 32, 33]. Although TEs comprise 11% (22.5 Mb) of the scaffolded *An. stephensi* genome, the most contiguous published draft assembly [18] contains 32% or 7.2 Mb less TE sequences, the majority of which (61% or 4.4 Mb) are composed of previously unknown LTR retrotransposons (Fig. 2a). The proportion of absent TE sequences in the draft assembly resembles the share of TE sequences missed by short reads-based TE detection [15]. Most full-length LTR and non-LTR retrotransposons we identified were either absent or fragmented in the existing draft genome assembly (Fig. 2a, Additional file 1: Figure S2, Additional file 2: Table S2). Some of these TEs are likely strain-specific and are absent from the strain sequenced previously [18]. The newly identified TEs include species-specific and evolutionarily recent retrotransposons, which highlight the dynamic landscape of new TEs in this species and provide a resource for modeling the spread of synthetic drive elements (Fig. 2b). The *An. stephensi* genome possesses fewer TEs and satellites than *An. gambiae*, partly accounting for the difference in their genome size and composition of the pericentric regions (Fig. 1a, Additional file 1: Figures S2 and S3). The difference in repeat density between the two species is particularly prominent on the X chromosome, which also carries disproportionately higher abundance of TEs and satellites among the three major chromosomes (*p* < 2.2e−16, proportion test for equal TE content) (Fig. 1a, Additional file 1: Figure S2). Although most (98% of 24.8 Mb) TEs are located within introns and intergenic sequences [34], we observed 1368 TE sequences in transcripts of 8381 Iso-Seq supported genes, 68% (939/1368) of which were not found in the earlier assembly [18] (Additional file 3: Table S3).

Due to the low, but measurable, level of residual heterozygosity in the sequenced strain (Additional file 1: Figure S1; “Materials and methods” section), we discovered several segregating TEs (Additional file 1: Figure S6, Additional file 4: Table S4), some of which likely have functional consequences. For example, a 975-bp LTR fragment inserted immediately at the 5′ end of the *Enolase* gene (*Eno*) may perturb its transcription (Fig. 2c). In *D. melanogaster*, null mutants of *Eno* show severe fitness and phenotypic defects that range from flightlessness to lethality [35], whereas reduction in *Eno* expression protects *Drosophila* from cadmium and lead toxicity [36]. Because *Eno* is highly conserved between *An. stephensi* and *D. melanogaster* (88% of 441 amino acids are identical between the two), we anticipate that this structural variant (SV) allele of *Eno* might be deleterious, although it also could confer some degree of resistance to heavy metal toxins. Another TE, a 874-bp DNA element, is inserted into the 3′ UTR of the gene *germ cell-less* (*gcl*), the *Drosophila* ortholog of which determines germ cell development [37] (Fig. 2d). All full-length Iso-Seq reads from this gene are from the non-insertion allele, suggesting that the insertion allele is a *gcl* null.

### Structural features and newly discovered genes of the Y chromosome

Targeting Y-linked sequences can be the basis for suppressing vector populations [38]. We identified 33 putative Y contigs using a k-mer-based approach (Fig. 1d). We experimentally validated three unique sequences spread across three contigs using PCR, which confirmed the male specificity of the predicted Y sequences (see the “Materials and methods” section). In contrast to the autosomes and the X chromosome, 72% of the Y sequences (1.7 Mb) comprise LTR elements (Fig. 2e, Additional file 1: Figure S6). While most full-length LTR elements in the Y chromosome also are present in the other chromosomes, a 3.7-kb retrotransposon, AST301, is represented by 46 highly similar (< 2.5% divergent) full-length copies only in the Y sequences. Its matching sequences elsewhere in the genome are vestigial and evolutionary distant, consistent with AST301 being primarily active in the Y chromosome (Fig. 2f). The proliferation of AST301 may be a consequence of the Y chromosome’s lack of recombination, which can lead to irreversible acquisition of deleterious mutations in a process called Muller’s ratchet [39].

Despite the high repeat content, we uncovered seven Y-linked genes that are supported by multiple uniquely mapping Iso-Seq reads (Additional file 1: Figure S7, Additional file 5: Table S5). We also recovered the three previously identified Y-linked genes and filled sequence gaps in *Syg1* [40]. Two of the newly discovered Y-linked genes (*Syg7* and *Syg8*) sit in a cluster of three overlapping Y-linked genes, all of which show strong expression in male larvae and adults but no expression in larval or adult females (Fig. 1, e and f). Y-linkage of these genes is also confirmed by PCR (see the “Materials and methods” section). Both genes show low or absent expression in the early (0–2 h) embryos but are expressed in the later stages (> 4 h) (Additional file 1: Figure S7). Translation of open reading frames from *Syg7* transcripts shows the presence of a myb/SANT-like domain in Adf-1 (MADF) domain in the encoded protein (Additional file 1: Figure S7).

### Transcriptional response to blood feeding

**Fig. 3:**
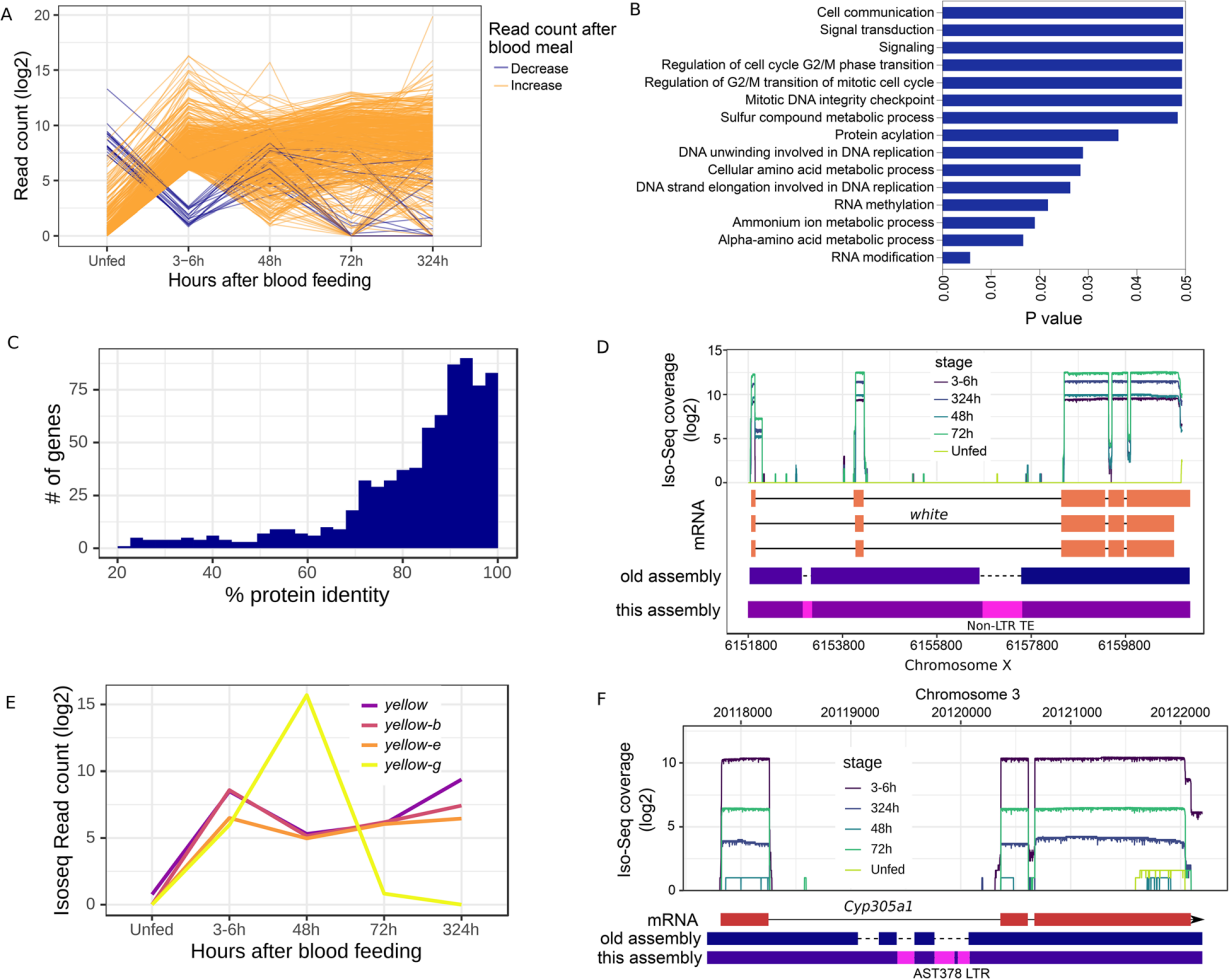
Gene expression changes in adult female mosquitoes after a blood meal. **a** Transcript abundance of genes that are in the top 1% (> ~ 64-fold) of the PBM transcript abundance changes. As evident here, more genes show upregulation than downregulation, although expression changes of some genes may not be due to the blood meal. **b** GO gene enrichment analysis of the genes from panel **a**. Consistent with the role of the blood meal in mosquito biology, the genes involved in cell division, DNA replication, amino acid metabolism, and cell signaling are enriched among the differentially expressed genes. **c** Protein sequence identity between the *An. stephensi* genes showing PBM upregulation and their *An. gambiae* orthologs. **d** Despite being a common genetic marker, the sequence of the PBM upregulated *white* gene was fragmented in the draft assembly of *An. stephensi*. **e** Transcript abundance of four *yellow* genes (*yellow*, *yellow-b*, *yellow-e*, *yellow-g*) before and after a blood meal. All genes show a similar transcript profile until 6 h PBM, after which *yellow-g* transcripts become more abundant. **f** A *Cyp450* orthologous to *D. melanogaster Cyp305a1* shows PBM upregulation and harbors intronic TEs are absent in the Jiang et al. [18] assembly

An alternative to suppression schemes aimed at reducing mosquito numbers is the modification of mosquito populations to prevent them from serving as parasite vectors. Promoters induced in females by blood feeding can be repurposed to express effector molecules that impede malaria parasite transmission [41, 42]. Following a blood meal, hundreds of genes are induced, many of which stay upregulated for days after the blood meal (Fig. 3a, Additional file 6: Table S6). When ranked by expression-fold changes post blood meal (PBM), the top 1% of genes with most strongly affected expression (representing a > 64-fold change) include 593 genes enriched for involvement in DNA replication, cell division, amino acid metabolism, and signaling within and between cells (Fig. 3b). Comparison of protein sequences of the upregulated genes and their orthologs in *An. gambiae* suggest that most genes are relatively conserved (70% share > 80% identity) (Fig. 3c). This suggests that the genes likely retained similar functions in the two species. However, sequences of 19.3% (115/593) of these genes are either fragmented or unannotated in the draft *An. stephensi* assembly [18] (Additional file 7: Table S7). For example, the eye pigmentation gene *white*, a key genetic marker in a wide range of insects and showing upregulation following a blood meal, was fragmented in the draft assembly due to the presence of an 853-bp intronic non-LTR retrotransposon (Fig. 3d). Similarly, despite the active roles of blood-inducible and constitutively expressed protease genes encoding trypsins and chymotrypsins in digestion and metabolism of blood [43, 44], not all of their complete sequences in *An. stephensi* were known before this study (Additional file 7: Table S7).

A Yellow protein gene (*yellow-g*) that was shown previously to be essential for female reproduction in *An. gambiae* and was used as a target in a CRISPR gene drive [45] was found to be upregulated PBM. Yet, neither *yellow-g* nor the three other members of the Yellow protein gene family (*yellow-b*, *yellow-e*, *yellow*) that showed PBM elevated transcript levels were previously annotated (Fig. 3e) [44]. Although transcript levels of all four genes increase in tandem until 6 h PBM, the *yellow-g* transcript level continues to climb until 48 h PBM and then reverts to the pre-BM level (Fig. 3e). In contrast, the other three *yellow* genes maintain a similar transcript level even after 13 days (Fig. 3e). The PBM upregulation patterns of the four yellow genes in *An. stephensi* are consistent with roles in female reproduction, although *yellow-b*, *yellow-e*, and *yellow* are probably required longer than *yellow-g*. Interestingly, a Cytochrome P450 monooxygenase (Cyp450) gene, which bears similarity to *D. melanogaster Cyp305a1*, also was upregulated (Fig. 3f). *Cyp305a1* acts as an epoxidase in the juvenile hormone biosynthesis pathway and helps maintain intestinal progenitor cells in *D. melanogaster* [46]. PBM upregulation of *Cyp305a1* suggests that it may have a potential role in cellular homeostasis in the midgut after a blood meal [47].

The *cis*-regulatory elements of the blood-meal-inducible genes can be combined with antimicrobial peptide genes to explore new effector molecule candidates to block malaria parasite transmission [41]. The new assembly revealed 361 immune-related genes, 103 of which were either previously unknown or broken in the previous assembly [18]. Among the immune-related genes, 15 genes were upregulated and are among the top 1% PBM upregulated genes (Additional file 6: Table S6). The immunity genes also included 20 putative antimicrobial peptides (AMP), three of which (GENE_00013347, GENE_00002217, GENE_00011093) were not known before (Additional file 1: supplementary text, Additional file 1: Figure S8, Additional file 8: Table S8). GENE_00013347 encodes for *Cecropin-A*, ortholog of which in *An. gambiae* protects against infections to Gram-negative and Gram-positive bacteria, fungi, and yeasts [48] and the malaria parasite *Plasmodium berghei* [49]. The AMPs and other immunity genes we identified provide a rich arsenal of effector molecule candidates for transgenic interventions to impair *Plasmodium* transmission in *An. stephensi*.

### Insecticide resistance genes

In Asia and eastern Africa, *An. stephensi* populations show widespread resistance to dieldrin, DDT, malathion, and pyrethroids [50–52]. Insecticide resistance in these populations has been attributed to various cytochrome p450s, esterases, GABA receptors (*resistance to dieldrin* or *rdl*), and voltage-gated sodium channels (*knock-down resistance* or *kdr*) [53]. Frequently, amino acid changes in *rdl* and *kdr* and copy number increases of Cyp450s, esterase (Est), and glutathione S-transferases (*GST*) have been associated with the resistance phenotypes [51, 53].

**Fig. 4:**
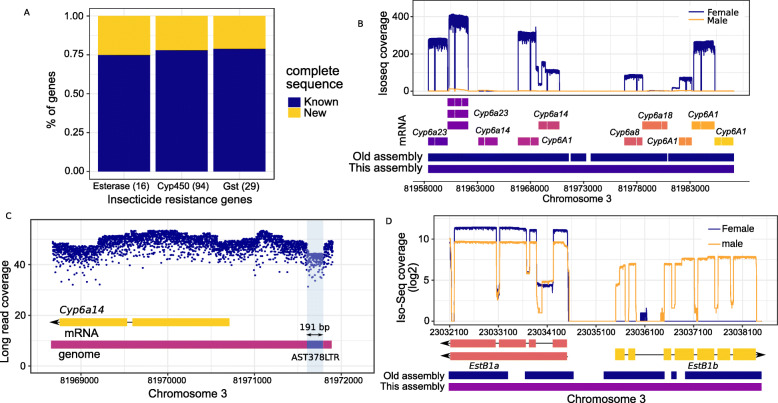
Putative insecticide resistance genes in *An. stephensi*. **a** Proportion of various candidate insecticide resistance genes that are either fragmented or missing repetitive regions in the draft *An. stephensi* assembly. **b** An array of tandemly located *Cyp450* genes that include *Cyp6a14*, a candidate gene for DDT resistance in *D. melanogaster* [54]. In the earlier assembly, this cluster was broken into three sequences, undermining investigation of the functional effects of these genes. Most genes show female-biased expression. **c** A polymorphic AST378 LTR fragment insertion is segregating inside the *Cyp450* array shown in B, suggesting the presence of more than one SV allele in this genomic region. **d** Tandemly located *Esterase B1* genes show different sex-biased expression patterns. *EstB1a* do not show any strong bias towards either sex, whereas *EstB1b* shows male-biased expression. *EstB1* amplification causes organophosphate resistance in *Culex.* The *EstB1a* and *EstB1b* sequences were broken into five pieces in the earlier *An. stephensi* assembly

We identified 94 Cyp450, 29 *GST*, and 16 esterase genes, including 2 acetylcholine esterases (*ace-1* and *ace-2*), providing a comprehensive resource for discovery and delineation of the molecular basis of insecticide resistance (see the “Materials and methods” section). Sequences of 22% (31/139) of these genes were either fragmented or missing in the draft assembly (see the “Materials and methods” section) (Fig. 4, a and b) [18]. We also recovered the complete *kdr* sequence and discovered 8 transcript isoforms of this gene (Additional file 1: Figure S9). A polymorphic TE insertion immediately downstream of *kdr* suggests that TE insertions play an important role in genetic variation in insecticide resistance candidate genes (Additional file 1: Figure S9). We also resolved tandem arrays of insecticide resistance genes, as evidenced by a 28-kb region consisting of Cyp450s similar to *D. melanogaster Cyp6a14*, *Cyp6a23*, *Cyp6a8*, *Cyp6a18*, and *Musca domestica Cyp6A1* (Fig. 4b). In *D. melanogaster*, *Cyp6a14* is a candidate gene for DDT resistance [54], suggesting a similar function for its *An. stephensi* counterpart. One *Cyp6a14* in the array has a polymorphic 191-bp LTR TE fragment inserted 1 kb upstream of the 5′ end of the transcription start site, implying the presence of more than one SV allele in this complex region (Fig. 4c). We also resolved previously fragmented tandem copies of *Esterase B1* (*Est-B1a* and *Est-B1b*) counterparts, which have been shown in *Culex quinquefasciatus* to provide resistance to organophosphates [55] (Fig. 4d). Interestingly, the Cyp450s in the array and *Est-B1b* show opposite sex-biased expression, suggesting that the molecular basis of insecticide resistance may differ between sexes (Fig. 4d).

## Discussion

Despite being an important malaria vector in Asia and an emerging vector in Africa, the existing genome assemblies of *An. stephensi* remain fragmented and incomplete. We improved the genetic resources for this species by assembling a highly contiguous de novo reference genome that recovers sequences relevant to the biology and evolution of the species missing in previous drafts. We found that the genome of *An. stephensi* is actively shaped by species-specific TEs, which likely comprise a major source of genetic variation. Given the generally known strongly deleterious effects of TE insertions [56, 57], the presence of potentially functionally relevant polymorphic TE insertions, even in an inbred laboratory strain, indicates that individually rare TE mutations could play a major role in the variation in phenotype and fitness of *An. stephensi* natural populations.

Even though the Y chromosome plays an important role in male biology and sexual conflicts, its highly repetitive nature poses steep challenges for assembly and molecular characterization of the Y chromosome in *Anopheles* [7, 8, 18, 28, 40, 58]. Due to the improved Y representation in our assembly, we characterized the repeat and gene content that previously evaded scrutiny. The enrichment and persistence of full-length LTR retrotransposons on this chromosome, relative to the autosome and the X chromosome, indicate an important role of these TEs in degeneration and heterochromatinization of the Y [18]. Moreover, we annotated at least seven previously undiscovered genes on the *An. stephensi* Y chromosome, suggesting that the paucity of Y-linked genes described in the *Anopheles* genus is due in part to technical limitations, rather than only the degeneration of the Y. While the biological functions of these genes remain unknown, one (*Syg7*) contains a MADF DNA-binding domain found in certain *D. melanogaster* transcription factors [59], suggesting it is a male-specific transcription factor. Thus, the Y sequences we uncovered provide insights into the contribution of Y towards the differences between the two sexes in *An. stephensi*.

In female *Anopheles* mosquitoes, blood meals initiate a cascade of physiological and molecular events involving hormone and signaling pathways, protein digestion and metabolism, gut homeostasis, and egg development. Our results uncovered several genes that show distinct patterns of regulation PBM, underscoring their respective roles in these processes. For example, persistence of the *yellow-g* transcript for days after a blood meal is consistent with its role in eggshell integrity in both *Drosophila* and *An. gambiae* [60, 61]. On the other hand, Cytochrome P450s, like *Cyp305a1,* play roles in hormone signaling and mediate intestinal homeostasis necessary for reproduction [62]. Thus, the new assembly, combined with the tracking of full-length transcripts, revealed the components of the complex biological network that are activated by a blood meal in this species. Further experiments using replicated transcript data will provide a comprehensive view of the effect of blood meal on *An. stephensi* biology.

The spread of insecticide resistance in Asian and African *An. stephensi* populations have made identification of insecticide-resistant mutations an urgent priority [50, 53, 63, 64]. We have uncovered TE insertions in the insecticide resistance gene *kdr*, which is generally investigated only for amino acid variants [65, 66]. TE insertions in the vicinity of a gene can influence its expression due to its epigenetic silencing effect on the expression of nearby genes [67]. The TE insertion at *kdr* could potentially affect *kdr* expression, contributing to functional variation that would go unnoticed by studies focusing on amino acid variation. Additionally, as we have shown here, candidate genes for insecticide resistance are often present in clusters of tandem copies that are difficult to resolve without a contiguous and error-free assembly. These regions are also segregating for repetitive structural variants (SV), indicating that SVs in repetitive genomic regions could contribute to functional genetic variation implicated in insecticide resistance in *An. stephensi* [68]. Evidently, such assemblies would be key to the detection of causal SV mutations for insecticide resistance [15, 20].

## Conclusion

CRISPR and gene drive-based strategies promise to transform the management of disease vectors and pest populations [69]. However, safety and effectiveness of these approaches rely on an accurate description of the functional and fitness effects of the genomic sequences and their variants [12, 70]. Draft assemblies are poorly suited for this purpose because they miss repetitive sequences or genes that are central to the vector’s biology and evolution. Incomplete information about the correct copies or sequence of a gene may mislead conclusions about functional significance of the gene or the target sequence [15] and may lead to mistargeting or misuse in a gene drive. The *An. stephensi* reference assembly mitigates these problems, revealing previously invisible or uncharacterized structural and functional genomic elements that shape various aspects of the vector biology of *An. stephensi*. Additionally, functionally important SVs are segregating even in this inbred lab stock, indicating a significant role of structural genetic variation in phenotypic variation in this species. Finally, recent advances in technology have sparked enthusiasm for sequencing all eukaryotes in the tree of life [21]. The assembly we report here is timely, as it constitutes the first malaria vector to reach the exacting reference standards called for by these ambitious proposals, and will stand alongside established references like those for human and fruit flies (Fig. 1b). This new assembly of *An. stephensi* provides a comprehensive and accurate map of genomic functional elements and will serve as a foundation for the new age of active genetics in *An. stephensi*.

## Materials and methods

### Mosquitoes

*Anopheles stephensi* mosquitoes of a strain (UCISS2018) from the Indian subcontinent (gift of M. Jacobs-Lorena, Johns Hopkins University) [71] were maintained in insectary conditions (27 °C and 77% humidity) with a photoperiod of 12 h light:12 h dark including 30 min of dawn and dusk at the University of California, Irvine (UCI). Larvae were reared in distilled water and fed ground TetraMin® fish food mixed with yeast powder. Adults had unlimited access to sucrose solutions (10% wt/vol) and females were provided with blood meals consisting of defibrinated calf blood (Colorado Serum Co., Denver) through the Hemotek® membrane feeding system. We established an isofemale line from the colony and inbred the line by sib mating for 5 generations prior to sequencing.

### Genome sequencing

Genomic DNA from 70 adult male and female mosquitoes was extracted using the Qiagen Blood & Cell Culture DNA Midi Kit following the previously described protocol [72]. The genomic DNA was sheared with 10 plunges of size 21 blunt needles, followed by 10 plunges of size 24 blunt end needles. We generated our PacBio reads using 31 SMRTcells on the RSII platform (P6-C4 chemistry) at the UC San Diego Genomics Core and 2 SMRTcells on Sequel I platform at Nucleome (Hyderabad, India). From the same genomic DNA, we also generated 3.7 GB of 300-bp paired-end reads at the UC San Diego genomics core and 32.37 GB of 150-bp paired-end Illumina reads from Nucleome. To identify the Y-linked contigs, we generated 27.8 GB and 28.5 GB 100 bp paired-end Illumina reads from male and female genomic DNA, respectively, at UCI Genomics High-Throughput Facility (GHTF).

### RNA extraction and sequencing

Total RNA was extracted from a total of six samples prepared from pooled individuals: 5–7-day-old sugar-fed males, 5–7-day-old sugar-fed females, and blood-fed females 3–6 h, 24 h, 48 h, and 72 h after feeding. The male pool consisted of 15 individuals, while female pools comprised 10 individuals each. All samples were isolated from the same mosquito cage. To do so, sugar-fed male and female samples were collected, and a blood meal offered for 1 h. Unfed females were removed from the cage and blood-fed females retrieved at each of the indicated time points. At the time of collection, samples were immersed in 500 μL of RNAlater RNA Stabilization Reagent (Qiagen) and stored at 4 °C. Total RNA was extracted using the RNeasy Mini Kit (Qiagen) following the manufacturer’s instructions for the Purification of Total RNA from Animal Tissues. Extracted samples were treated with DNA-free Kit (Ambion) to remove traces of genomic DNA. Finally, samples were cleaned using the RNA Clean & Concentrator Kit (Zymo Research). mRNA selection, cDNA synthesis, and Iso-Seq library prep were performed at UCI GHTF following the manufacturer’s (Pacific Biosciences) protocol. For each of the six samples, one SMRTcell of Iso-seq reads was generated on the Sequel I platform.

### Genome assembly

We used 42.4 GB or 180✕ of long reads (assuming haploid genome size G = 235 Mb) to generate two draft assemblies of *An. stephensi* using Canu v1.7 [73] and Falcon v2.1.4 [74]. Falcon was used to assemble the heterozygous regions (Additional file 1: Figure S1) that were recalcitrant to Canu. We filled the gaps in the Canu assembly using the Falcon primary contigs following the two-steps merging approach with Quickmerge v0.3, where the Canu assembly was used as the reference assembly in the first merging step [72, 75]. The resulting assembly was processed with finisherSC (v2.1) to remove the redundant contigs and to fill the further gaps with raw reads [76]. This PacBio assembly (613 contigs, contig N50 = 38.1 Mb, 257.1 Mb in total) was polished twice with Arrow (smrtanalysis v5.2.1) and twice with Pilon v1.22 using ~400X (80 Gb) 150 bp PE Illumina reads from our three Illumina datasets [77].

### Identification of polymorphic mutations

To identify the variants segregating in the sequenced strain, we aligned the alternate haplotype contigs (a_ctg.fa) identified by Falcon to the scaffolded assembly. Then we called the indels using SVMU v0.2 (Structural Variants from MUmmer). An indel was marked as a TE based on its overlap with the Repeatmasker annotated TEs. To estimate heterozygosity, we mapped the Illumina reads to the chromosome scaffolds using Bowtie2 (v2.2.7) and converted the alignments to a sorted bam file using SAMtools (v1.9). A VCF file containing the SNPs and small indels were generated using freebayes (v1.3.2-40-gcce27fc), and pairwise nucleotide diversity (pi) was calculated over 25-kb windows using vcftools (vcftools --window-pi 25,000; v0.1.14v0.1.14).

### Microbial sequence decontamination

Microbial contigs in the assembly were identified using Kraken v2.0.7-beta [78] (Additional file 1: supplementary text) [79–82], which assigned taxonomic labels to the 613 contigs (Additional file 1: Figure S4). Kraken mapped k-mers (31–35 nt default) from the 613 contig sequences against the databases from the six domain sets: bacteria, archaea, viral, UniVec_Core, fungi, and protozoa from National Center for Biotechnology Information (NCBI) and the genome sets including representative reference mosquito from VectorBase v2019-02 and *Drosophila* genomes (*n* = 24; Additional file 1: Table S9). The databases map k-mers to the lowest common ancestor (LCA) of all genomes known to contain a given k-mer. The Kraken label for each contig was further classified as either *Anopheles*, contaminating (non-*Anopheles*), or unclassified (no hit in the database) (Additional file 1: Figure S4). To prevent false positives in the results, low-complexity sequences in the assembly were masked with dustmasker (blast v2.8.1) [83] prior to running Kraken. The mitochondrial genome of *An. stephensi* was identified by aligning the existing mitogenome (GenBank No. KT899888) against the contigs using nucmer in MUMmer v4.0.0b [84].

### Scaffolding

To de novo scaffold the microbial decontaminated 566 contigs (Additional file 1: supplementary text) [85–89], we collected HiC data from adult male and female mosquitoes filled up to the 1 ml mark of a 1.5-ml Eppendorf tube. We flash-froze the adult mosquitoes and sent them to Arima Genomics (San Diego) to generate a HiC library using the Arima kit. This library was sequenced on a single flow cell of an Illumina HiSeq 2500 instrument, generating 326 GB of Illumina 150-bp paired-end reads. We mapped the HiC reads to the *An. stephensi* contigs using Juicer v1.5.6 [90] and used the resulting contact map to scaffold the contigs using 3D-DNA v180922 [91]. The order and orientation of the three chromosomes were examined by nucmer in MUMmer v4.0.0b alignment of 20 gene/probe physical map data (X, 5 probes; 2, 7; 3, 8) generated from fluorescence in situ hybridization (FISH) on polytene chromosomes (Additional file 1: Figure S10; Additional file 9: Table S10) [18] against Hi-C chromosome assemblies. The aligned probes showed 83–100% sequence identities to our assembly, confirming the chromosome nomenclature we used based on synteny (Additional file 1: Figure S10, Additional file 9: Table S10).

### QV estimation and assembly statistics

To estimate the error rate in our final assembly, we mapped the paired-end Illumina reads to the assembly using bwa mem (bwa v0.7.17-5). The alignments were converted to bam format and then sorted using SAMtools (v.1.8-11). We called the variants using freeBayes v0.9.21 [92] (command: freebayes -C 2 -0 -O -q 20 -z 0.10 -E 0 -X -u -p 2 -F 0.75) and followed the approach of [73] to calculate QV (10*-log10(2981/250,632,892)). Briefly, we counted the number of bases comprising homozygous variants in the assembly (2981) and then divided it by the total mapped bases that had a coverage of at least three (250,632,892). We used QUAST v5.0.3 to obtain assembly statistics [93]. Out of 250 Mb, 205 Mb (82%) was scaffolded into the three chromosome-length scaffolds that correspond to the three *An. stephensi* chromosomes (chrX, 22.7 Mb; chr2, 93.7 Mb; chr3, 88.7 Mb). We identified 66 unplaced contigs as alternate haplotigs (66 contigs = 7.2 Mb) using mummer alignments of contigs to the major chromosomes. Additionally, 103 (8.6 Mb) of 458 (35.9 Mb) unplaced or unclassified contigs were identified as alternate haplotigs using BUSCO v4.1.4 Diptera odb10 dataset [23] and the software Purge_dups v1.0 [94] (Table 1) (Additional file 10: Table S12). The final Hi-C map was visualized using HiCExplorer v3.4.2 [95]. We estimated the proportion of scaffolded haploid genomes using the publicly available resources on *An. stephensi* and *An. gambiae* genome size [96, 97]. The *C* value of *An. stephensi* is 0.24 and that of *An. gambiae* is 0.27. Based on the odds ratio of the genome sizes of the two species estimated from their *C* values and the *An. gambiae* genome size (265 Mb), we inferred the genome size for *An. stephensi* to be ~ 235 Mb.

### Repeat annotation

We created a custom TE library using the EDTA (Extensive *de-novo* TE Annotator) pipeline [98] and Repeatmodeler v2.0.0 (http://www.repeatmasker.org/RepeatModeler/) to annotate the TEs. LTR retrotransposons and DNA elements were identified de novo using EDTA, but because EDTA does not identify non-LTR retrotransposons, Repeatmodeler was used to identify these. The two libraries were combined and the final library was used with Repeatmasker (v4.0.7) to annotate the genome-wide TEs. Tandem repeats were annotated using Tandem Repeat Finder v4.09 [99]. The number and copy number of micro-, mini-, and macro-satellites spanning in each 100-kb non-overlapping window of the three chromosomes were identified. The satellite classification was made as described in [18]. In brief, tandem repeats were classified as micro- (1–6 bases), mini- (7–99 bases), and macro- (≥ 100 bases) satellites. Mini- and macro-satellites were considered only if they had a copy number of more than 2. All these three simple repeats were considered only if they had at least 80% sequence identity, and set some cutoff (≥ 2 copy number; ≥ 80% identity) to screen high confidence repeats, then the overall abundance was calculated.

### Annotation using Iso-Seq

In total, six samples (5 females, 1 male) of *An. stephensi* mosquitoes were used for Iso-Seq sequencing (see the “RNA extraction and sequencing” section). Raw PacBio long-molecule sequencing data was processed using the SMRT analysis v7.0.0 Iso-Seq3 pipeline [100]. Briefly, CCS was used to generate the full-length (FL) reads for which all 5′-end primer, polyA tail, and 3′-end primer have been sequenced and then Lima was used to identify and remove the 5′- and 3′-end cDNA primers from the FL reads. The resulting bam files were processed with Iso-Seq3 to refine and cluster the reads, which were polished with Arrow. This de novo pipeline outputs FASTQ files containing two sets of error-corrected, full-length isoforms: (i) the high-quality set contains isoforms supported by at least two FL reads with an accuracy of at least 99% and (ii) the low-quality set contains isoforms with an accuracy < 99% that occurred due to insufficient coverage or rare transcripts. The high-quality isoforms were collapsed with Cupcake v10.0.1 and were used in Talon v5.0 for annotation [101]. We combined the high-quality isoforms with other lines of evidence using MAKER2 v2.31.10 to create a final annotation (see below).

### MAKER annotation

The final annotation of the genome was performed using MAKER2 v2.31.10 [102], which combines empirical evidence and ab initio gene prediction to produce final annotations. We used MAKER2 for three cycles of gene predictions. First, the Iso-Seq data were used as evidence for training MAKER2 for gene predictions. We also used transcriptome and peptide sequence data from *An. gambiae* (PEST4.12) and *An. funestus* (FUMOZ 3.1) as alternative evidence to support the predicted gene models. Prior to gene annotation, repeats were masked using RepeatMasker included in MAKER2. Mapping of EST and protein evidence to the genome by MAKER2 using BLASTn and BLASTx, respectively, yielded 12,324 genes transcribing 14,888 mRNAs. The output of first round gene models were used for the second round, where MAKER2 ran SNAP and AUGUSTUS for ab initio gene predictions. Next, another round of SNAP and AUGUSTUS predictions were performed to synthesize the final annotations that produced 14,966 genes, transcribing 16,559 mRNAs. In total, we identified 56,388 exons, 9791 5′-end UTRs, 9290 3′end UTRs, and 503 tRNAs (Table 1; see the “Materials and methods” section). We also predicted ab initio an additional 14,192 mRNAs/proteins but due to weak support they were not considered. The final MAKER annotation was assessed using recommended AED and Pfam statistical metrics.

The gene models were functionally annotated in MAKER2 v2.31.10 through a homology BLAST search to UniProt-Sprot database, while the domains in the annotated proteins were assigned from the InterProScan database (Additional file 1: supplementary text). We compared our gene model annotations with the draft assembly using OrthoFinder v2.3.7 [103] The GO enrichment analysis was performed in PANTHER v15.0 using PANTHER GO-SLIM Biological Process annotation data set [104]. Further, the orthologous top 1% of *An. stephensi* upregulated gene protein sequences in *An. gambiae* were identified by OrthoFinder v2.3.7.

### Validation and quantification with RNAseq and Iso-Seq

To quantify transcript abundance using Iso-seq reads, raw reads were mapped to the genome assembly using minimap2 [105] and the gene-specific transcript abundance was measured using bedtools, requiring that each Iso-seq read overlaps at least 75% of gene length annotated with TALON v5.0 (bedtools coverage -mean -F 0.75 -a talon.gff -b minimap.bam) [106]. To take variation due to sequencing yield per SMRTcell into account while calculating transcript abundance, Iso-seq coverage of each gene was divided by a normalization factor, which was calculated by dividing the total read counts for each sample by the total read counts from the unfed female sample. To identify the genes up- or downregulated due to blood feeding, we compared the transcript abundance of 5–7-day-old adult females before and after blood meal. To identify the genes whose expressions are most strongly affected by blood meal, we rank-ordered transcript abundance differences for all genes showing non-zero transcript abundance in either of the two samples. Here we reported the genes that are in the top 1% of the transcript level differences which corresponds to all genes showing > ~ 64-fold increase or decrease in transcript abundance between the two samples.

To obtain transcript levels of Y-linked genes from embryos, larvae, and adults, we used publicly available RNA-seq data (Additional file 1: Table S11). RNA-seq reads were mapped to the genome using HISAT2, and the per-base read coverage was calculated from the sorted bam files using samtools depth. Additionally, the bam files were processed with stringtie to generate sample-specific transcript annotation in GTF format [107]. Sample-specific GTF files were merged with stringtie to generate the final GTF. To obtain the gene model and transcript isoforms of *kdr*, stringtie annotated transcripts that covered the entire predicted ORF based on homology with *D. melanogaster para* [108] were used.

### Identification of new genes and repeat elements

To identify incomplete or absent sequences in the most contiguous *An. stephensi* published assembly [18], the contigs from the draft assembly were aligned to the new assembly using nucmer [84] and alignments due to repeats were filtered using delta-filter to generate 1-to-1 mapping (delta-filter -r -q) between the two assemblies. The resulting delta file was converted into tab-separated alignment format using show-cords utility in MUMmer (v4). To identify TE sequences that are present in our assembly but absent in Jiang et al. (2014), we annotated the TEs in the latter using RepeatMasker and calculated the abundance of different classes of TEs (DNA, LTR, Long Interspersed Nuclear Elements or non-LTR) from the RepeatMasker output. Additionally, we identified TE sequences in our assembly that were either fragmented or absent in the Jiang et al. [18] contig assembly by looking for TE sequences that either failed to map or mapped only partially to the latter (bedtools intersect -v -f 1.0 -a te.bed -b alignment.bed). To identify the genes that were fragmented or absent from the Jiang et al. [18] data, we combined two complementary approaches. First, we used the 1-to-1 genome alignment to identify genes that were split over > 1 contig. Second, we mapped the annotated protein sequences from our study to the protein sequences reported in Jiang et al. using OrthoFinder v.2.3.7 [103] and identified the transcripts that were present only in our study. We combined the unique genes found by each approach to calculate the total number of previously incomplete or missing genes. To rule out assembly errors in the new assembly as the cause of discrepancy between the two assemblies, 20 features disagreeing between the assemblies were randomly selected from each category and manually inspected in IGV. At least 3 long reads spanning an entire feature was used as evidence for correct assembly of the features.

### Identification of Y contigs

To identify the putative Y contigs, male- and female-specific k-mers were identified from male and female paired-end Illumina reads using Jellyfish [109] (Additional file 1: Figure S11). The density of male and female k-mers for each contig was calculated, and the contigs showing more than twofold higher density of male-specific k-mers were designated as putative Y contigs. Interestingly, the *Serratia* genome we assembled also showed similar male k-mer enrichment as the Y contigs.

### Experimental validation of Y-linked contigs

The k-mer-based approach employed to identify male-specific kmers that occur at a rate 20-fold higher than female-specific kmers in the *An. stephensi* scaffolds (Additional file 1: Figure S11). In order to verify putative Y-linked sequences, ten 2–3 day-old male or female *An. stephensi* mosquitoes per replicate were used for the experiment. Genomic DNA was extracted from each sample using DNeasy Blood and Tissue Kit (Cat # 69504). Gene-specific primers (Y15 forward (F)—ATT TTA GTT ATT TAG AGG CTT CGA, Y15 reverse (R)—GCG TAT GAT AGA AAC CGC AT; Y22 F—ATG CCA AAA AAA CGG TTG CG, Y22 R—CTA GCT CTT GTA AAG AGT CAC CTT; Y28 F—ATG CTA CAA AAC AGT GCC TT, Y28 R—TTA GGT CAG ATA TAG ACA CAG ACA CA) were designed based on the genome sequence to amplify ≥ 500-bp products using polymerase chain reaction (PCR) reaction. The amplification was done using Q5 high fidelity 2X Master Mix (Cat # M0492). Amplicons were resolved in agarose gels and male versus female amplification was compared. The PCR products were gel eluted and Sanger sequenced (Additional file 1: Figure S11) (Genewiz) with forward PCR primer. The identity of the sequencing was confirmed by aligning the amplicon sequences against the *An. stephensi* genome assembly using BLAST.

### Identification of putative immune gene families

Studying the patterns of evolution in innate immune genes facilitate understanding the evolutionary dynamics of *An. stephensi* and pathogens they harbor. A total of 1649 manually curated immune proteins of *An. gambiae* (Agam 385), *Ae. aegypti* (Aaeg 422), *Cu. quinquefasciatus* (Cpip 495) and *D. melanogaster* (Dmel 347) in ImmunoDB (Additional file 8: Table S8) [110] were used as databases to search for the putative immune-related proteins in MAKER2-annotated protein sequences of the *An. stephensi* assembly using sequence alignment and phylogenetic orthology inference-based method in OrthoFinder v2.3.7. The number of single copy orthogroup/orthologous proteins (one-to-one) and co-orthologous and paralogous proteins in *An. stephensi* were identified (one-to-many; many-to-one; many-to-many) (Additional file 1: supplementary text) [111].

## Supplementary Information

**Additional file 1: Figure S1.** Assembly coverage and heterozygosity. **Figure S2.** Distribution of repeats and estimates of new full length TEs. **Figure S3.** Tandem repeats in *An. stephensi*. **Figure S4.** Identification and annotation of microbial sequences. **Figure S5.** Comparison of assembly contiguity and completeness of genome assemblies of various mosquitoes and *D. melanogaster*. **Figure S6.** TE insertion in a functional gene and TE content of *An. stephensi* chromosomes. **Figure S7.** Y-linked genes supported by uniquely mapping Iso-Seq reads. **Figure S8.** The repertoire of putative immune-related proteins of *An. stephensi* that belong to 27 gene families. **Figure S9.** *kdr* gene of *An. stephensi* and presence of SV near the gene. **Figure S10.** Position of 20 physical map probes against their sequence identity to the new *An. stephensi* genome assembly. **Figure S11.** Identification and validation of Y-linked sequences. **Table S1.** Comparison of assembly statistics for *An. stephensi* older and new assemblies. **Table S9.** A list of 23 mosquito genomes and *D. melanogaster* reference genome from VectorBase/NCBI that were used to create a custom database for Kraken2 to classify *An. stephensi* contigs. **Table S11.** SRA accession of the publicly available RNAseq data used in this study.

**Additional file 2: Table S2.** Coordinates of TE sequences that were not found in the existing draft assembly of *An. stephensi*.

**Additional file 3: Table S3.** Coordinates of exonic TE sequences that were not found in the existing draft assembly of *An. stephensi*.

**Additional file 4: Table S4.** Coordinates of polymorphic TE sequences that are present in the scaffolds assigned to chromosomes but absent in the alternate haplotype sequences.

**Additional file 5: Table S5** Coordinates of putative Y-linked genes supported by multiple uniquely mapping Iso-Seq reads.

**Additional file 6: Table S6.** Genes that are in the top 1% (>64 fold) category of the up- or down-regulated genes after blood feeding.

**Additional file 7: Table S7.** PBM up or down regulated genes that are either fragmented and missing repetitive sequences like TEs and tandem copies.

**Additional file 8: Table S8.** Classification of putative immune-related proteins of *An. stephensi*.

**Additional file 9: Table S10.** Details of physical map data used in this study.

**Additional file 10: Table S12.** List of 103 unclassified contigs identified as alternate haplotigs using BUSCO Diptera data set and the software Purge_dups.

## Data Availability

The sequenced strain is available at no cost from AAJ. The raw PacBio, Illumina, and Hi-C sequencing data and *An. stephensi* genome assembly were deposited in the NCBI BioProject database (accession number PRJNA629843) [112]. The annotations and other genomic features can be accessed at http://3.93.125.130/tigs/anstephdb/ [113]. The final MAKER2 GFF file, the list of insecticide resistance genes, the list of immunity genes, and the list of novel transcripts are available at https://github.com/mahulchak/stephensi_genome [114]. All codes used in the study, including those used to make figures, are available at https://github.com/mahulchak/stephensi_genome [114].
