# Author Correction: Hidden genomic features of an invasive malaria vector, *Anopheles stephensi*, revealed by a chromosome-level genome assembly

**DOI:** 10.1186/s12915-022-01314-2

**Published:** 2022-05-02

**Authors:** Mahul Chakraborty, Arunachalam Ramaiah, Adriana Adolfi, Paige Halas, Bhagyashree Kaduskar, Luna Thanh Ngo, Suvratha Jayaprasad, Kiran Paul, Saurabh Whadgar, Subhashini Srinivasan, Suresh Subramani, Ethan Bier, Anthony A. James, J. J. Emerson

**Affiliations:** 1 Department of Ecology and Evolutionary Biology, University of California, Irvine, CA 92697 USA; 2 Section of Cell and Developmental Biology, University of California, La Jolla, San Diego, CA 92093-0335 USA; 3 Tata Institute for Genetics and Society, Center at inStem, Bangalore, Karnataka 560065 India; 4 Department of Microbiology & Molecular Genetics, University of California, Irvine, CA 92697 USA; 5 Institute of Bioinformatics and Applied Biotechnology, Bangalore, KA 560100 India; 6 Section of Molecular Biology, University of California, La Jolla, San Diego, CA 92093-0322 USA; 7 Tata Institute for Genetics and Society, University of California, La Jolla, San Diego, CA 92093-0335 USA; 8 Department of Molecular Biology & Biochemistry, University of California, Irvine, CA 92697 USA; 9 Center for Complex Biological Systems, University of California, Irvine, CA 92697 USA

**Correction to: BMC Biol 19, 1-16 (2021)**

**https://doi.org/10.1186/s12915-021-00963-z**

The original article [1] contained an error in Fig. 3 and omitted a Funding source which the authors would like to correct.

Due to a labeling error in one of our Iso-Seq samples, the RNA sample that was collected 24h after blood feeding was labeled as 324 by the sequencing center because they unknowingly removed a separator between the replicate number and the sample name. The error resulted in a different ordering of categories than we would have chosen, though this doesn’t actually affect the interpretations we made in the manuscript. The corrected Fig. 3 can be viewed ahead in this correction article.

**Fig. 3:**
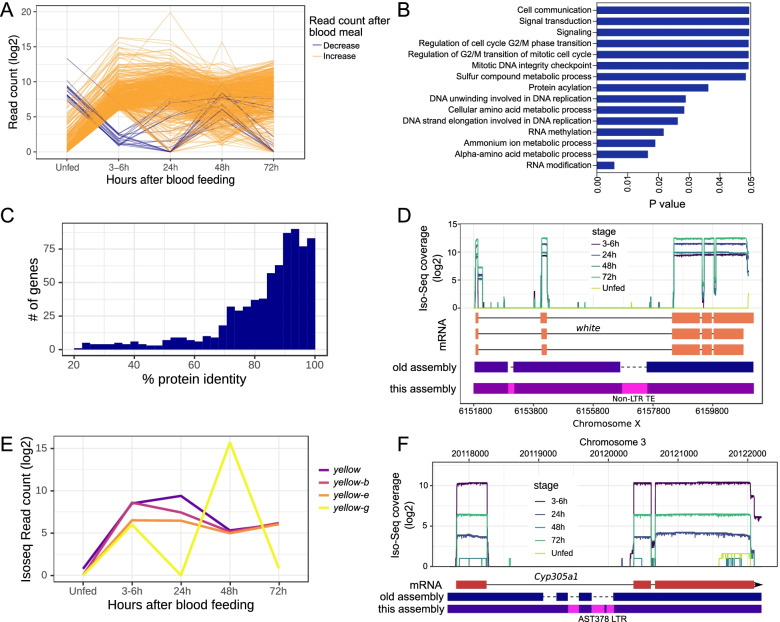
Gene expression changes in adult female mosquitoes after a blood meal. **a** Transcript abundance of genes that are in the top 1% (> ~ 64-fold) of the PBM transcript abundance changes. As evident here, more genes show upregulation than downregulation, although expression changes of some genes may not be due to the blood meal. **b** GO gene enrichment analysis of the genes from panel **a**. Consistent with the role of the blood meal in mosquito biology, the genes involved in cell division, DNA replication, amino acid metabolism, and cell signaling are enriched among the differentially expressed genes. **c** Protein sequence identity between the *An. stephensi* genes showing PBM upregulation and their *An. gambiae* orthologs. **d** Despite being a common genetic marker, the sequence of the PBM upregulated *white* gene was fragmented in the draft assembly of *An. stephensi*. **e** Transcript abundance of four *yellow* genes (*yellow*, *yellow-b*, *yellow-e*, *yellow-g*) before and after a blood meal. All genes show a similar transcript profile until 6 h PBM, after which *yellow-g* transcripts become more abundant. **f** A *Cyp450* orthologous to *D. melanogaster Cyp305a1* shows PBM upregulation and harbors intronic TEs are absent in the Jiang et al. [18] assembly

Although the error does not affect any conclusion, it remains technically inaccurate and merits correction.

We also did not acknowledge funding from the United States National Science Foundation (NSF) to J.J.E. for development of the sex chromosome inference approach. This is an important oversight, as NSF requires acknowledgment. The grant number is: NSF grant IOS-1656260.

## References

[1] Chakraborty M (2021). Hidden genomic features of an invasive malaria vector, Anopheles stephensi, revealed by a chromosome-level genome assembly. BMC Biol.

