# Supplementary material for: Hidden genomic features of an invasive malaria vector, Anopheles stephensi, revealed by a chromosome-level genome assembly
Source: BMC Biol. 2021 Feb 10;19:28. doi: 10.1186/s12915-021-00963-z (PMC7876825; doi:10.1186/s12915-021-00963-z)
Supplement: Supplementary file 1 — Additional file 1: Figure S1. Assembly coverage and heterozygosity. Figure S2. Distribution of repeats and estimates of new full length TEs. Figure S3. Tandem repeats in An. stephensi. Figure S4. Identification and annotation of microbial sequences. Figure S5. Comparison of assembly contiguity and completeness of genome assemblies of various mosquitoes and D. melanogaster. Figure S6. TE insertion in a functional gene and TE content of An. stephensi chromosomes. Figure S7. Y-linked genes supported by uniquely mapping Iso-Seq reads. Figure S8. The repertoire of putative immune-related proteins of An. stephensi that belong to 27 gene families. Figure S9. kdr gene of An. stephensi and presence of SV near the gene. Figure S10. Position of 20 physical map probes against their sequence identity to the new An. stephensi genome assembly. Figure S11. Identification and validation of Y-linked sequences. Table S1. Comparison of assembly statistics for An. stephensi older and new assemblies. Table S9. A list of 23 mosquito genomes and D. melanogaster reference genome from VectorBase/NCBI that were used to create a custom database for Kraken2 to classify An. stephensi contigs. Table S11. SRA accession of the publicly available RNAseq data used in this study. [file 12915_2021_963_MOESM1_ESM.pdf]

Supplementary materials

Hidden genomic features of an invasive malaria vector, *Anopheles stephensi*, revealed by a chromosome-level genome assembly

Mahul Chakraborty[1*], Arunachalam Ramaiah[1,2,3*], Adriana Adolfi[4], Paige Halas[4], Bhagyashree Kaduskar[2,3], Luna Thanh Ngo[1], Suvratha Jayaprasad[5], Kiran Paul[5], Saurabh Whadgar[5], Subhashini Srinivasan[3,5], Suresh Subramani[3,6,7], Ethan Bier[2,7], Anthony A. James[4,7,8], J.J. Emerson[1,9,#]

[1]Department of Ecology and Evolutionary Biology, University of California, Irvine, CA 92697, USA
[2]Section of Cell and Developmental Biology, University of California, San Diego, La Jolla, CA 92093-0335, USA
[3]Tata Institute for Genetics and Society, Center at inStem, Bangalore, Karnataka 560065, India
[4]Department of Microbiology & Molecular Genetics, University of California, Irvine, CA 92697, USA
[5]Institute of Bioinformatics and Applied Biotechnology, Bangalore, KA 560100, India
[6]Section of Molecular Biology, University of California, San Diego, La Jolla, CA 92093-0322, USA
[7]Tata Institute for Genetics and Society, University of California, San Diego, La Jolla, CA 92093-0335, USA
[8]Department of Molecular Biology & Biochemistry, University of California, Irvine, CA 92697, USA
[9]Center for Complex Biological Systems, University of California, Irvine, CA 92697, USA

*These authors contributed equally to this work

[#]Correspondence to: J.J. Emerson, jje@uci.edu

This Additional file includes:

Supplementary text

Figures S1 to S11

Tables S1, S9, and S11

Supplementary text

Assessment of genome completeness by BUSCO

The BUSCO based universal 3285 orthologs (Diptera database) analysis showed the completeness of the reference-quality *An. stephensi* genome (99.7%) that we assembled exceeds the previous assemblies (draft assembly 97.2%; Astel2 98.9%) (Additional file 1: Fig. S5). Comparison with the recent version of chromosome-level genome assemblies of other *Anopheles* species (range 97-99.6%), *Aedes aegypti* (97.6%)*, Culex quinquefasciatus* (87.7%) and *Drosophila melanogaster* (99.5%) showed that the completeness of *An. stephensi* genome exceeds all these assemblies (Additional file 1: Fig. S5). Among the 3285 Diptera BUSCOs in *An. stephensi* assembly, 3276 (99.7%) were complete (3256 (99.2%) were single-copy; 18 (0.5%) were duplicated), 2 fragmented and 7 are missing BUSCOs. All complete BUSCOs were identified in major 3 chromosomes, except one in unclassified contig (ucontig283). Among the 18 duplicated BUSCOs in our assembly, 12 BUSCOs were identified within or between the major three chromosomes.

We compared BUSCOs of our *An. stephensi* assembly with well studied *An. gambiae* reference assembly (Additional file 1: Fig. S5). A total of 3178 single-copy BUSCOs were common in *An. stephensi* and *An. gambiae*. There were 68 single-copy BUSCOs identified in *An. stephensi* were duplicated in *An. gambiae*, while 18 single-copy BUSCOs in *An. gambiae* were duplicated in *An. stephensi*. Totally, 12 (13499at7147, 19449at7147, 20465at7147, 22971at7147, 31040at7147, 40537at7147, 49088at7147, 57159at7147, 59859at7147, 60890at7147, 62774at7147, 70323at7147) and 5 (24608at7147, 45582at7147, 70842at7147, 74791at7147, 78504at7147) single-copy BUSCOs were unique to *An. stephensi* and *An. gamabie*, respectively. One duplicated BUSCO (33706at7147) was unique to *An. gambiae*.

Microbial and mitochondrial sequences

An. stephensi genomic DNA was expected to contain microbial DNA from endosymbionts, lab contaminations, and environment. In total, 8% of the contigs (46/613) were from microbial sources and one contig (1/613) represented the mitochondrial genome (Additional file 1: Fig. S4). Complications from assembling a circular genome using softwares specializing on *de novo* assembly of linear genomes created three tandem copies of the mitochondrial genome in a 45 Kb contig. Manual trimming and curation produced a single complete mitogenome (15 Kb) that shared 99.8% sequence identity with a Chinese *An. stephensi* mitogenome (GenBank #KT899888.1). Among the 46 microbial contigs, 45 (1.3 Kb – 5.58 Mb) belonged to 12 bacterial species of three phyla: proteobacteria (37), bacteroidetes (7) and actinobacteria (1). Interestingly, the complete genomes of facultative bacteria *Serratia marcescens* (5.58 Mb in total; 3 contigs) and a double stranded DNA virus *Salmonella phage* (60.2 Kb, 1 contig) were assembled. These three *Serratia* contigs were scaffolded into a first circular complete genome of the facultative endosymbiont *Serratia marcescens* from *Anopheles* (Additional file 1: Fig. S4). The members of the Proteobacteria *Serratia* and *Asaia* and Flavobacteria *Elizabethkingia* were also common among the microbial contigs. These are also found in *Anopheles gambiae*. This *Salmonella phage* virus genome has GC content of 58%, which is genetically similar to the virus reported from sewage samples or sewage-contaminated river water samples from India (GenBank ID: KR296691).

Immune-related genes/proteins

Using curated sets of immune-related proteins, a total of 361 immune genes (31, chrX; 159, chr2; 142, chr3; 11, alternate haplotypes; 18, unclassified contigs) transcribing 394 putative immunity transcripts/proteins that were belonging to 27 gene families were identified in adult *An. stephensi* mosquitoes (Additional file 8: Table S8). The list of these genes are also available on GitHub (see Data and materials availability). None of

them were identified from putative Y-linked contigs. Expansion of many protein families (AMP, APHAG, CLIP, CTL, ML, SCR, SRPN, SRRP, IMDPATH and TOLLPATH) relative to *An. gambiae* account for the large *An. stephensi* immunity-related gene repertoire (Additional file 1: Fig. S8; Additional file 8: Table S8). Among 394 proteins, 221 (orthologous to Agam, 205; Aaeg, 8; Cpip, 7; Dmel, 1) were identified as single-copy orthogroup proteins (Additional file 1: Fig. S8). Out of the remaining 173 proteins, 51 proteins are in a one-to-many relationship, 97 proteins are in many-to-one relationships, and 25 proteins are in a many-to-many relationship (possibly due to gene duplication events) with the known immune proteins. Interestingly, a total of 16 proteins in CLIP, ML, PRDX, SRPN and SRRP families have also been identified to share orthologous proteins with distantly related two mosquitoes Aaeg and Cpip, and Dmel. Our findings showed that the majority of the (i.e. 93% of single-gene) immune proteins were found to share orthologous proteins with *An. gambaie*. Protein expansion in signal modulation IMDPATH family was possibly due to the presence of gram-negative bacteria including symbiont *S. marcescens* (Additional file 8: Table S8). It also indicated that the rate of gene duplication was higher in *An. stephensi* than in *An. gambiae*. Among 394 immune-related transcripts the top three most abundant transcripts represent signal modulation CLIPs and SRRPs, and recognition CTLs (72, 46 and 30 respectively). Gene losses were observed in families FREPs, GALEs and PPOs.

Supplementary tables

Table S1. Comparison of assembly statistics for *An. stephensi* older and new assemblies.

Features	Old assembly *(9)*	This assembly
Total length (bp)	221,309,404	250,632,892
Contig number	31,761	566
Contig N50	36,511	38,117,870
Scaffold number	23,371	560^
Scaffold N50	1,591,355	88,747,609*
L50	40	2
GC content (%)	44.8	44.91

^Except three major chromosomes, we kept others as contigs; *Scaffold N50 is the length of chr3

Table S9. A list of 23 mosquito genomes and *D. melanogaster* reference genome from VectorBase/NCBI that were used to create a custom database for Kraken2 to classify *An. stephensi* contigs.

Species Name	Strain	# Scaffolds/Chromosome
Aedes aegypti	LVP_AGWG	3
Aedes albopictus	Foshan	154782
Anopheles albimanus	ALBI9	5
Anopheles arabiensis	DONG5	1214
Anopheles atroparvus	EBRO	582
Anopheles christyi	ACHKN1017	30369
Anopheles coluzzii	M	10521
Anopheles culicifacies	A-37	5230
Anopheles darlingi	COARI	2220
Anopheles dirus	WRAIR2	1266
Anopheles epiroticus	epiroticus2	2673
Anopheles farauti	FAR1	116
Anopheles gambiae	PEST	5
Anopheles maculatus	maculatus3	5556
Anopheles melas	CM1001059_A	5723
Anopheles merus	MAF	1027
Anopheles minimus	MINIMUS1	678
Anopheles quadriannulatus	QUAD4	2823
Anopheles sinensis	China	8007
Anopheles sinensis	SINENSIS	3101
Anopheles stephensi	Indian_wild_type	526
Anopheles stephensi	SDA-500	1110
Culex pipiens quinquefasciatus	Johannesburg	3171

Drosophila melanogaster	A4	7

Table S11. SRA accession of the publicly available RNAseq data used in this study.

Sample type	SRA ID
adult female	SRR1851030, SRR1851028, SRR1851027, SRR515307
adult male	SRR1851026, SRR1851024, SRR1851022, SRR515308
female larvae	SRR8156253, SRR8156254, SRR8156255, SRR8156256
0-1h embryo	SRR7061580, SRR7061576
2-4h embryo	SRR7061579, SRR7061575
4-8h embryo	SRR7061578, SRR7061574
8-12h embryo	SRR7061577, SRR7061573

Supplementary Figures

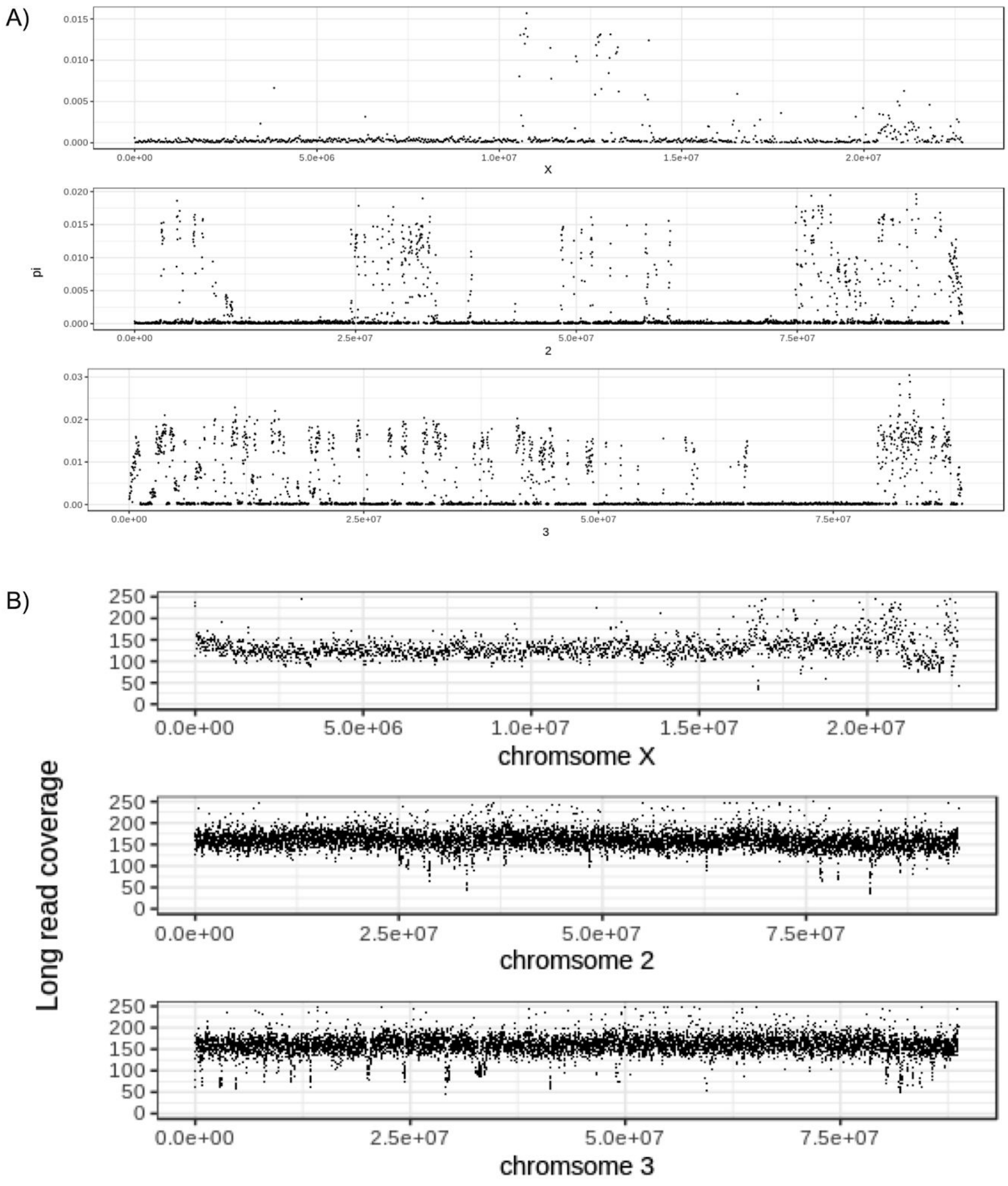

Figure S1. Assembly coverage and heterozygosity. (**A**) Heterozygosity across the major chromosome arms (X,2,3) of the inbred sequenced Indian strain of *An. stephensi*. As evidenced here, chromosome 3 has more residual heterozygosity than the other

chromosomes. (**B**) Long read coverage in 100bp windows across the scaffolded major chromosome arm sequences. Intermittent coverage drops to non-zero values indicate presence of >1 haplotype in that region. Consistent with the chromosome 3 harboring the highest amount of heterozygosity (refer panel A), such coverage drops are most common in the 3rd chromosome.

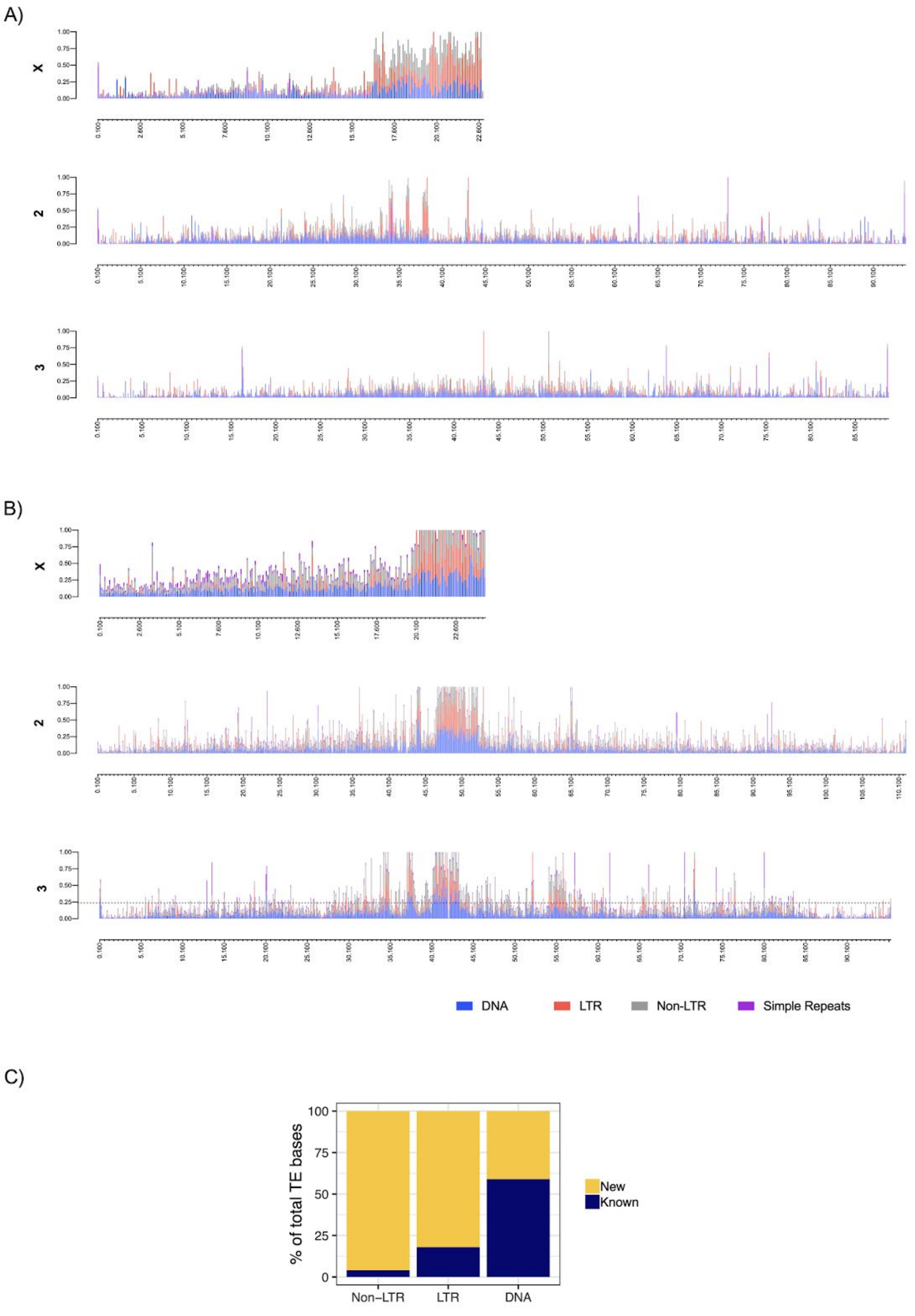

Figure S2. Distribution of repeats and estimates of new full length TEs. The repeat content across the three chromosomes in (**A**) *An. stephensi* and (**B**) *An. gambiae*. The repeat content in the genome was estimated using RepeatMasker and Tandem Repeat

Finder. Each bar represents the proportion of different repeat types in 100 Kb non-overlapping windows indicate that the density of repeats on the sex chromosome X is more than that of the autosomes. (**C**) Proportion of TEs (counted in bp) that are present in the new *An. stephensi* assembly but fragmented or absent (new) in the draft assembly.

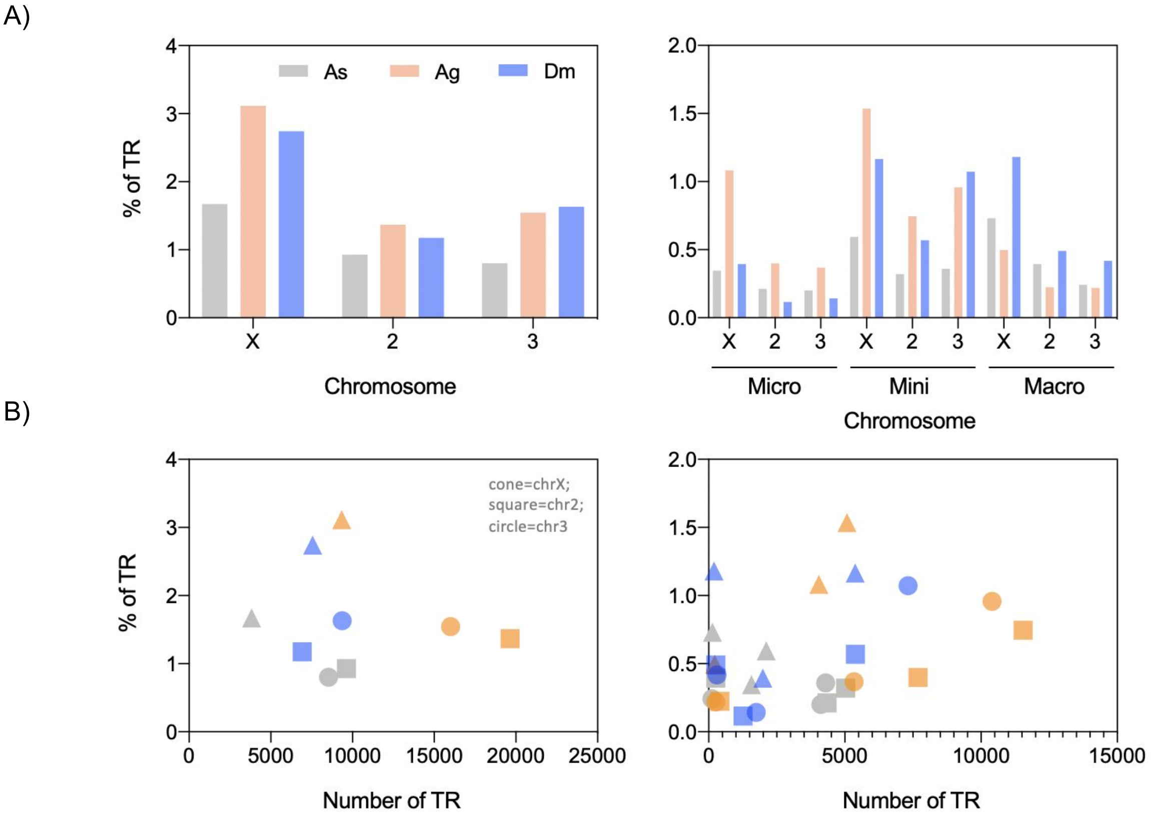

Figure S3. Tandem repeats in *An. stephensi*. (**A**) A comparison of the simple tandem repeats (TR) abundance in three chromosomes of *An. stephensi* (As, grey), *An. gambiae* (Ag, orange) and *D. melanogaster* (Dm, blue) identified using Tandem Repeats Finder. (**B**) The actual number of repeats scattered against the proportion of TR (normalized length of TR). TR consists of a combination of the micro, mini and

macrosatellites that were shown in the right panel of the A and B. The proportion of the simple repeats is higher in *An. gambiae* than in *An. stephensi* and *D. melanogaster*.

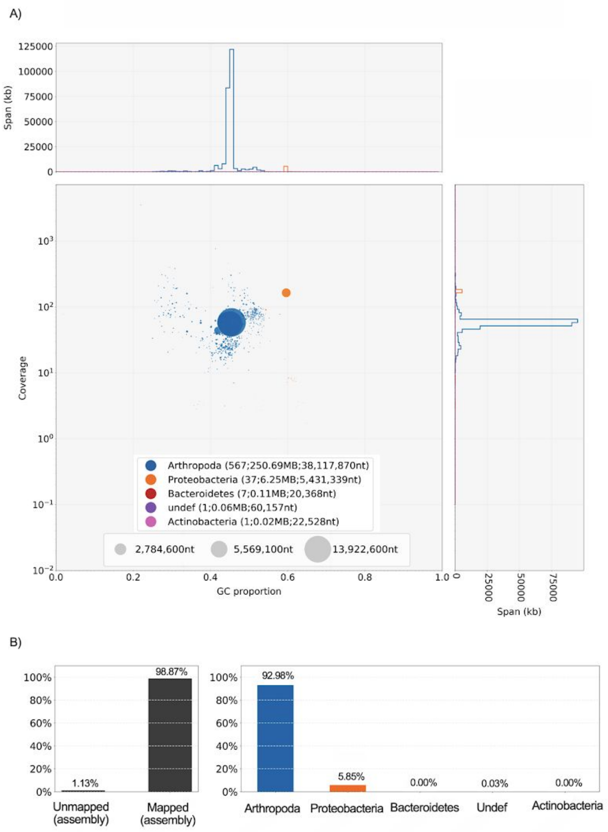

C)

Species Name	NCBI Taxonomy	# Contigs	Total Nt bp
Aedes aegypti	7159	1	45214
Anopheles dirus	7168	1	25886
Anopheles stephensi	30069	565	250615945
Asaia bogorensis	1231624	16	402452
Burkholderia cepacia	1395570	3	27608
Citrobacter koseri	545	1	22321
Elizabethkingia anophelis	1352940/1338011	7	105341
Klebsiella oxytoca	571	1	1334
Komagataeibacter saccharivorans	265959	1	15936
Microbacterium sp.	2048898	1	22528
Pandoraea sputorum	93222	5	78571
Pseudomonas sp.	286	1	14547
Rahnella aquatilis	745277/1151116	2	36811
Rahnella sp.	1805933	4	73025
Serratia marcescens	615 /1334564 / 1401254	3	5582294
Salmonella phage	1654890	1	60157

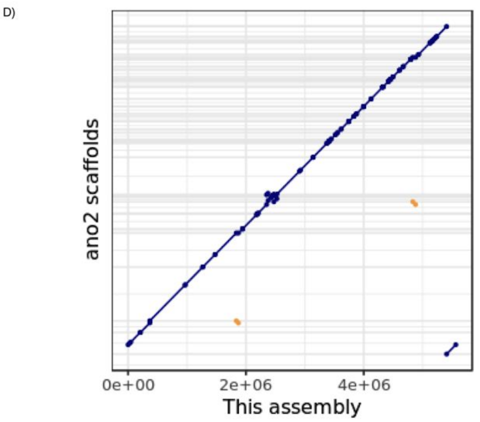

Figure S4. Identification and annotation of microbial sequences. Taxonomy classification of contigs from whole-genome assembly of *An. stephensi* using BlobTools. (**A**) Blobplot shows base coverage in a read set of whole-genome sequencing against GC content for contigs. Contigs are colored by phylum with Arthropoda (blue), Proteobacteria (orange), Bacteroides (red) and Actinobacteria (pink). A single contig classified as a DNA virus (purple). Histograms show the distribution of contig length sum along each axis. (**B**) Proportion of classified contigs. (**C**) A total of 92.98% of 613 contigs are classified as Arthropoda, while the remaining 46 are microbial contigs (6.4 Mb) that belong to 12 bacteria and one DNA phage virus. (**D**) Dot plot between *Serratia*

marcescens assembly from this study (X axis) and the most contiguous strain of *An. stephensi S. marcescens* (ano2) from NCBI. As evident from the plot, the new assembly has the entire *S. marcescens* genome in a single contig, whereas ano2 has 77 scaffolds. Notably, several structural differences exist between ano2 and new reference strain.

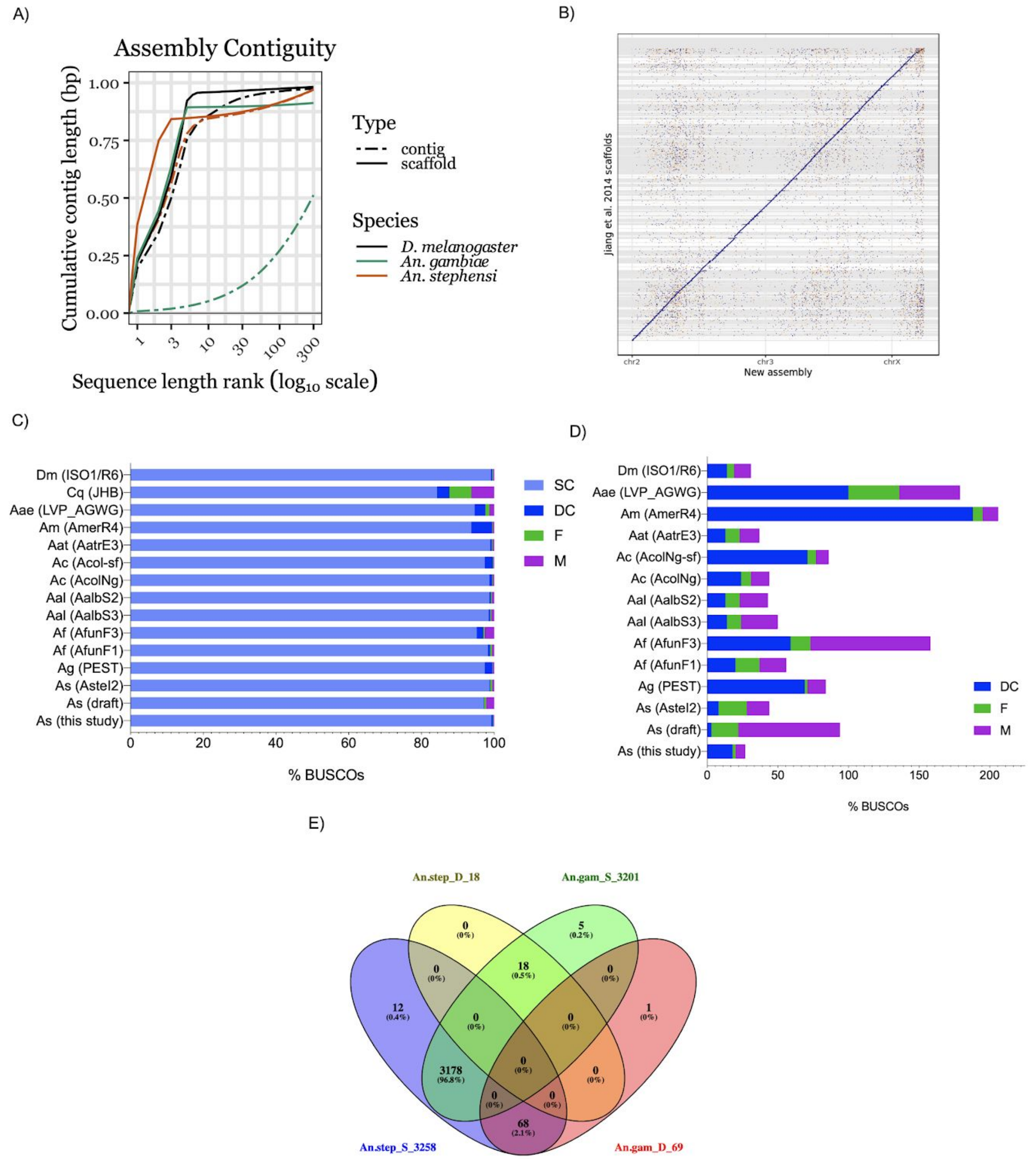

Figure S5. Comparison of assembly contiguity and completeness of genome assemblies of various mosquitoes and *D. melanogaster*. (**A**) Comparison of assembly contiguities between *An. stephensi*, *An. gambiae*, and *D. melanogaster* reference assemblies. (**B**) Dot plot between the new reference assembly of *An. stephensi* and the

Jiang et al. 2014 draft-quality assembly, Each horizontal line denotes scaffold boundary from the older assembly and each vertical line denotes scaffold boundaries of the assembly from this study. The diagonal demonstrates overall concordance between the two assemblies. The presence of densely positioned numerous horizontal lines (appearing as grey shaded rectangles) demonstrates fragmentation of the older assembly. (**C**) Diptera lineage Benchmarking Universal Single-Copy Orthologs (BUSCO; n=3285) assessment was used to quantify completeness for *An. stephensi* (As; new, draft and Astel2 assemblies), along with the latest version of published chromosome-level assemblies of *An. gambiae* (Ag), *An. funestus* (Af), *An. albimanus* (Aal)*, An. coluzzii* (Ac), *An. atroparvus* (Aat), *An. merus* (Am), *Aedes aegypti* (Aae), *Culex quinquefasciatus* (Cq), and *Drosophila melanogaster* (Dm) genomes. It showed that *An. stephensi* new assembly is a best characterized genome among the sequenced malaria vectors. Bar charts show proportions classified as complete C - complete (SC, Single-copy complete; DC, Duplicated complete), F - fragmented and M - missing. (**D**) Comparison of the number of duplicated (D), fragmented (F) and missing (M) BUSCOs among the species (except Cq) shown in A. (**E**) Comparison of BUSCOs of the new *An. stephensi* (An.step) assembly and *An. gambiae* (An.gam). Singleton (An.step_S; An.gam_S) and Duplicated (An.step_D; An.gam_D) BUSCOs were compared to identify common and unique BUSCOs. The number of BUSCOs identified in both species under two categories was also labelled in the venn diagram.

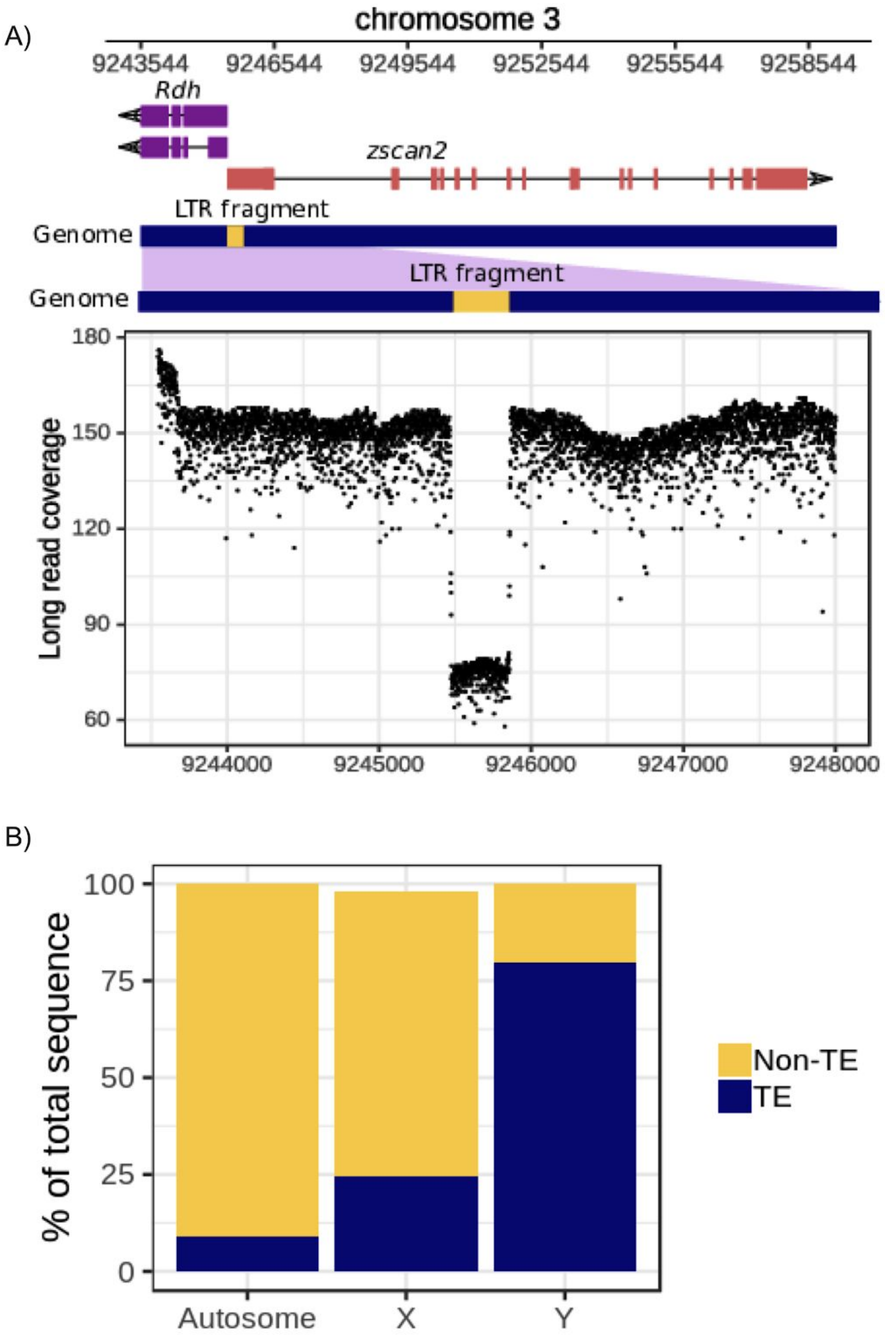

Figure S6. TE insertion in a functional gene and TE content of *An. stephensi* chromosomes. (**A**) Polymorphic insertion of a LTR TE fragment within the first exon of *zscan2* and immediately upstream of retinaldehyde dehydrogenase (*Rdh*). The coverage drop to nearly 50% (coverage ~75) for the LTR fragment suggests presence of the insertion in only half of the 3rd chromosomes segregating in the strain. Given that

promoters and cis-regulatory sites are often located immediately upstream of a gene, this polymorphic TE insertion could influence transcription of these two genes. (**B**) Proportion of TE bases in assembled sequences of autosomes (2nd and 3rd), X, and Y. X has more TEs than autosomes but Y has the greatest proportion, with 78% of the Y sequences being TEs.

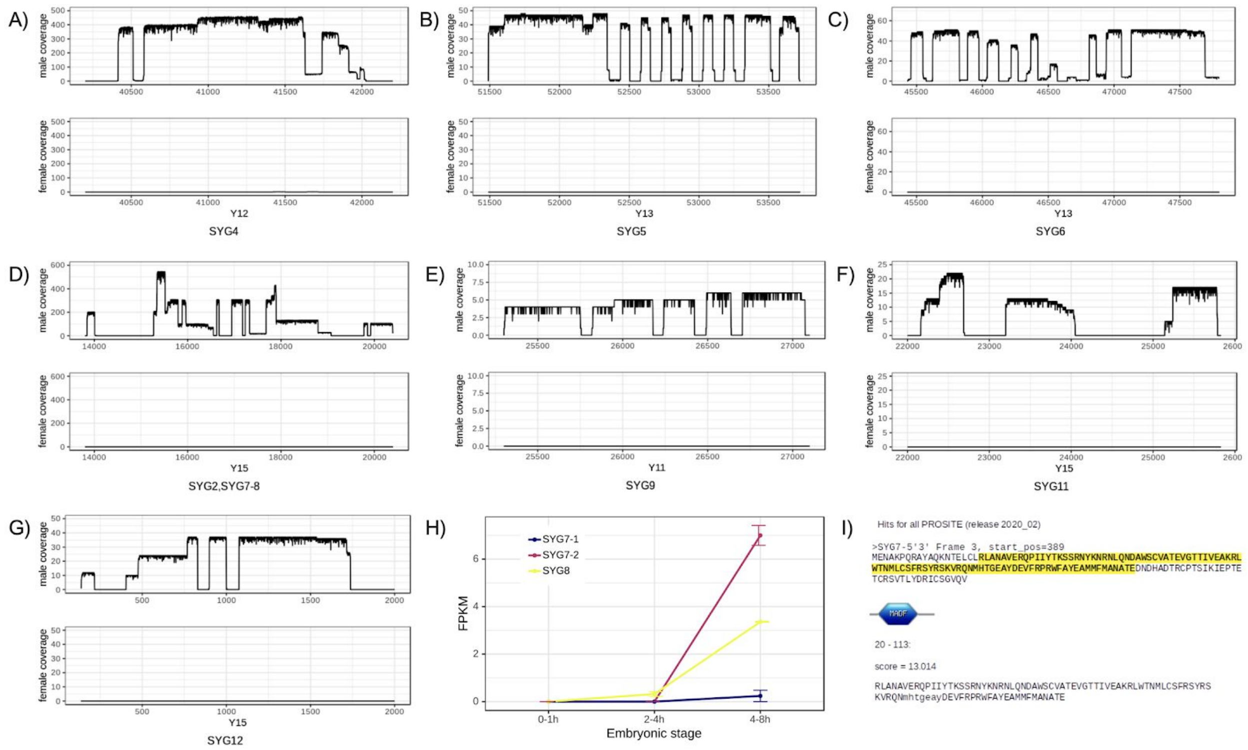

Figure S7. Y-linked genes supported by uniquely mapping Iso-Seq reads. Supporting evidence for Y-linked gene (**A**) SYG4, (**B**) SYG5, (**C**) SYG6, (**D**) SYG2, SYG7, SYG8 (detailed gene models of SYG2, SYG7, and SYG8 are depicted in Fig. 1E), (**E**) SYG9, (**F**) SYG11 (the two levels of coverages in the first exon of the gene are due to two transcript isoforms; see table S5), and (**G**) SYG12 (due to the partial fragmentation of the full length mRNA, the 5' end of the transcript (left side of the plot) has lower coverage than the 3' end) from Isoseq read coverage. Exons have more or less uniform coverage from Isoseq reads collected from adult male mRNA, whereas introns are represented by large coverage drops. Consistent with the Y-linkage of this gene, no Isoseq read from adult females map to it. (**H**) Expression of *SYG7* and *SYG8* in early embryos, where both begin to be expressed after 4 hours. (**I**) Presence of MADF domain in the translated protein sequence from *SYG7* transcript. The transcript sequence predicted by Iso-Seq reads were translated with an expasy protein translation tool and then scanned with PROSITE.

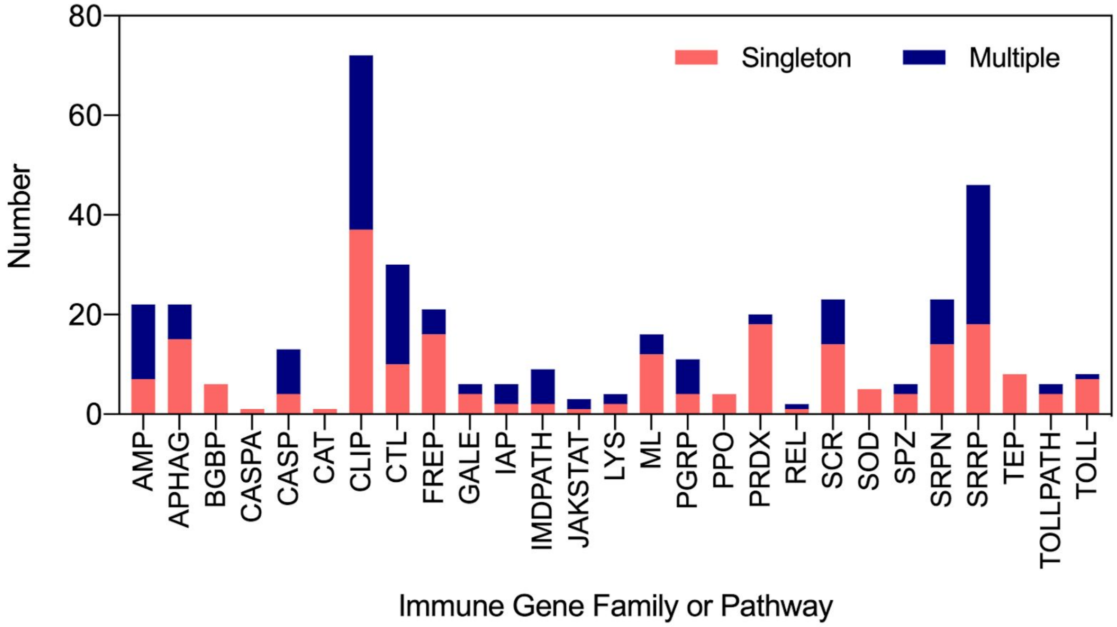

Figure S8. The repertoire of putative immune-related proteins of *An. stephensi* that belong to 27 gene families. Among 394 proteins, 221 are identified as single-copy (orange) while remaining 173 proteins (blue) are identified to have one-to-many, many-to-one and many-to-many (blue) relationships with the curated proteins from the immune database (Additional file 8: Table S8).

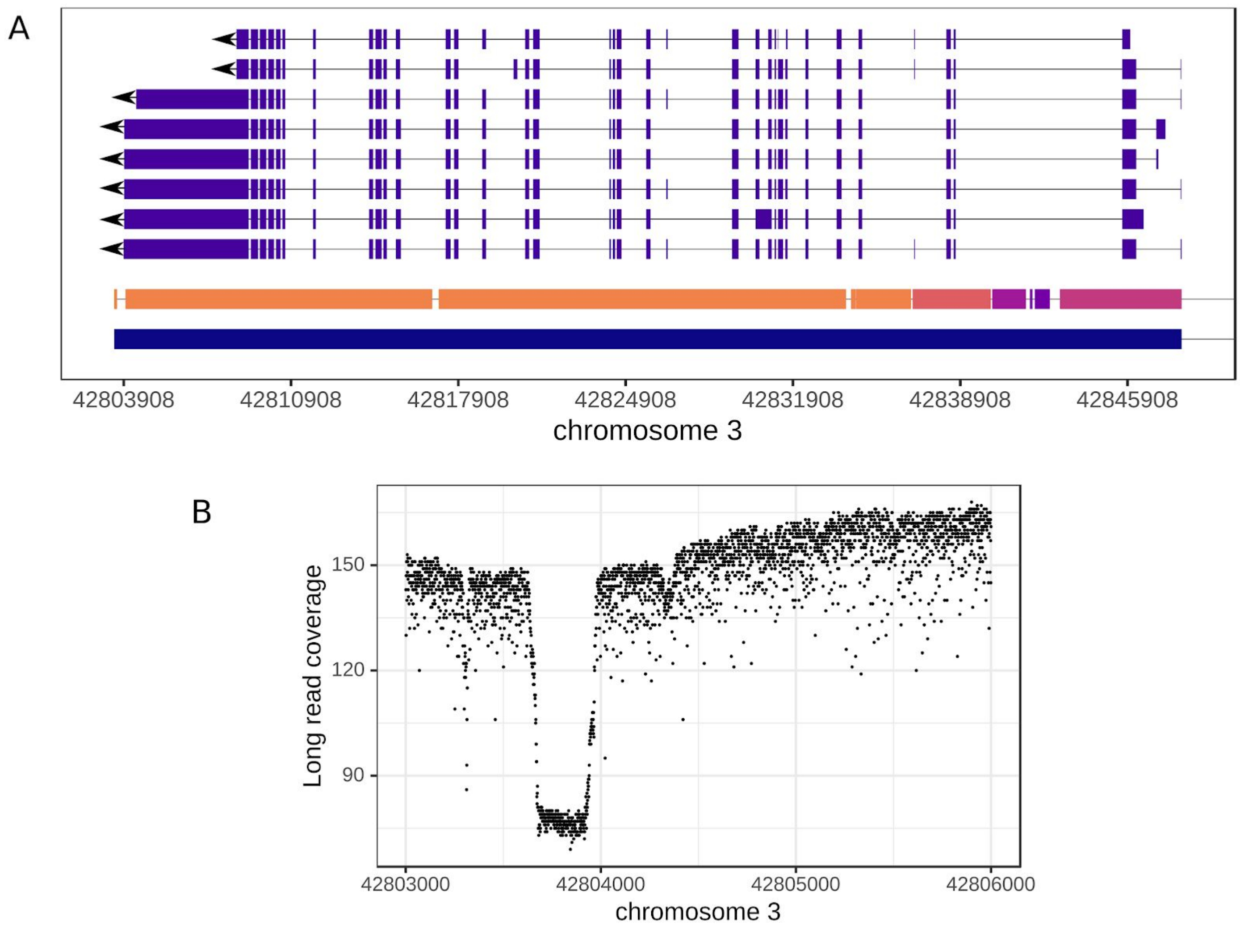

Figure S9. *kdr* gene of *An. stephensi* and presence of SV near the gene. (**A**) Multiple transcript isoforms of *kdr* easily detected using RNAseq reads mapped to the new *An. stephensi* reference assembly (solid blue bar). However, the older draft assembly has the *kdr* gene split over 6 contigs (different colors of bars above the blue solid bar represents contigs in the older assembly). (**B**) A polymorphic indel immediately downstream of the *kdr* gene, providing evidence that SVs are segregating in this candidate insecticide resistance gene in this strain. Evidence of the indel can be seen as the leftmost gap in the older assembly in A.

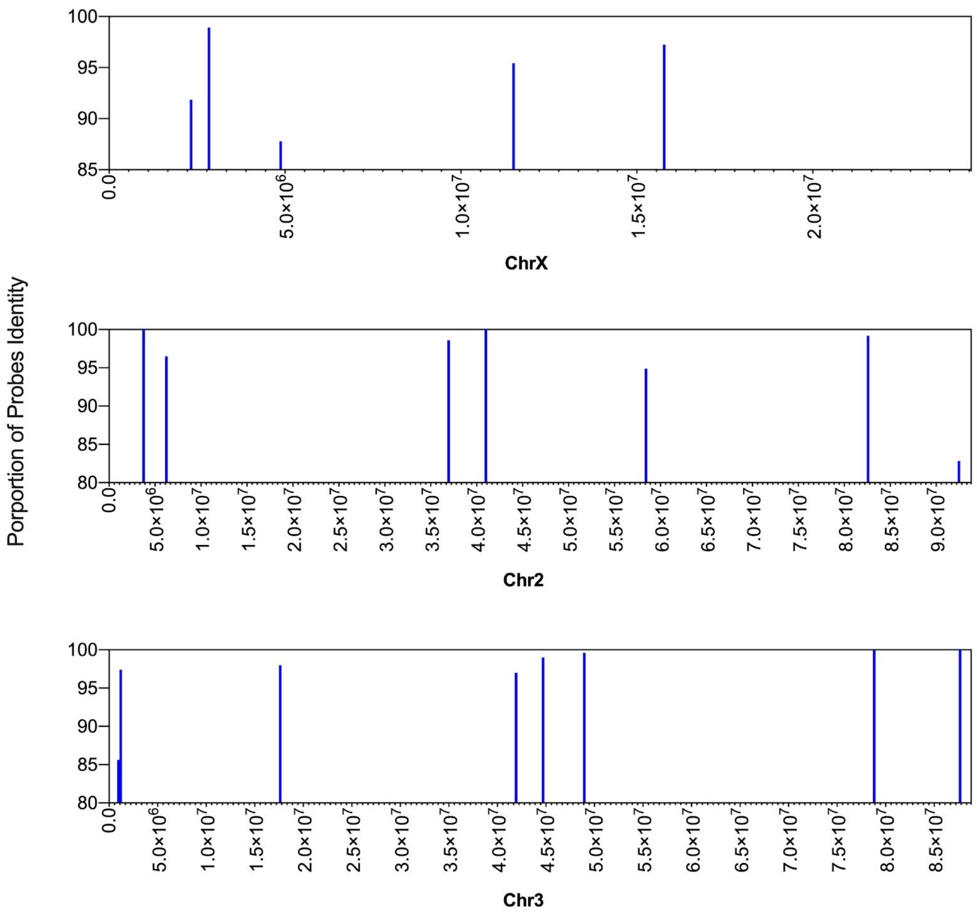

Figure S10. Position of 20 physical map probes against their sequence identity to the new *An. stephensi* genome assembly. The order and orientation of the three chromosomes are examined by MUMmer alignment of 20 gene/probe physical map data (chrX, 5 probes; chr2, 7; chr3, 8) generated from FISH on polytene chromosomes (Additional file 9: Table S10) against Hi-C chromosome assemblies.

A)

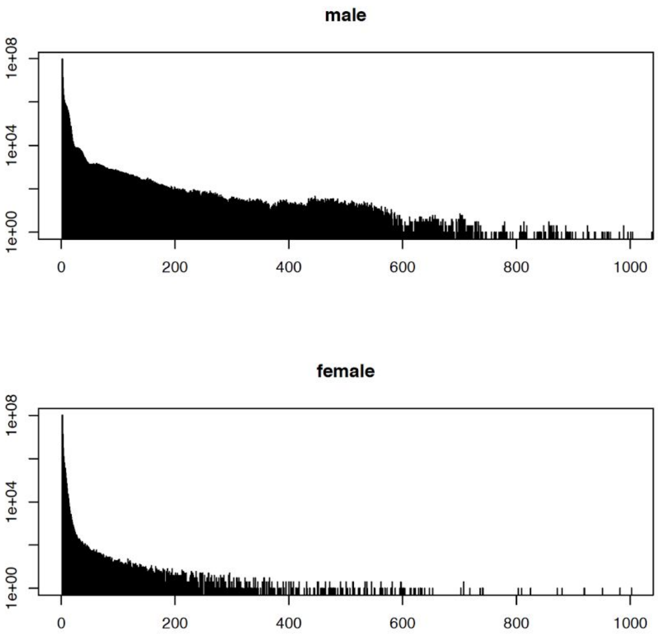

B)

chrY15	maker	mRNA	15336	19330	+	ANSTEP-UCI_TRAN_00014237-RB	Parent=ANSTEP-UCI_GENE_00014237	Ma5_7d	Protein of unknown function
chrY22	maker	mRNA	149789	150373	+	ANSTEP-UCI_TRAN_00013933-RA	Parent=ANSTEP-UCI_GENE_00013933		Protein of unknown function
chrY28	maker	mRNA	6145	19836	-	ANSTEP-UCI_TRAN_00014965-RA	Parent=ANSTEP-UCI_GENE_00014965		Similar to Bbx: HMG box transcription factor BBX (Mus musculus OX%3D10090)

C)

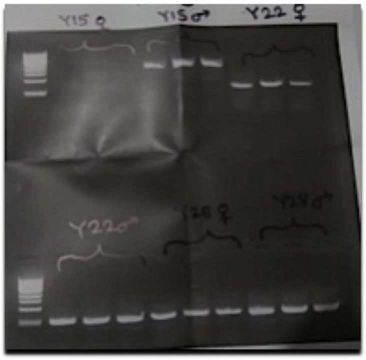

D)

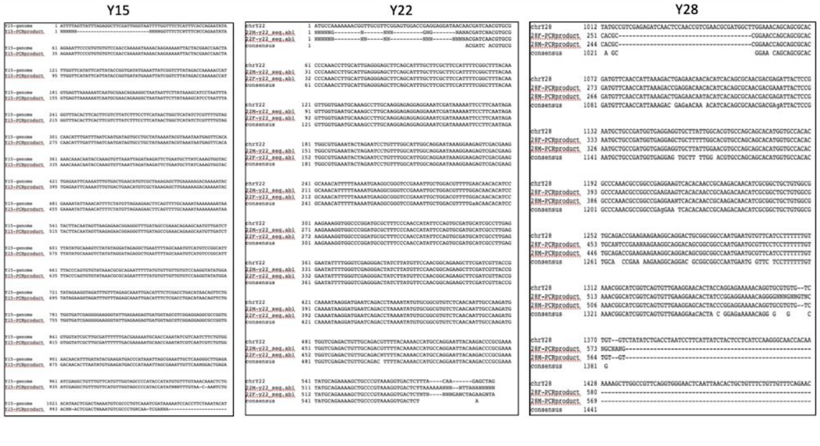

Figure S11. Identification and validation of Y-linked sequences. (**A**) Male and female specific k-mers identified from the paired-end Illumina reads that were generated from the male (top) and female (bottom) genomic DNA, respectively. (**B**) Experimental validation of putative Y chromosome contigs. Three genomic regions of putative Y contigs that were examined with PCR. These regions also correspond to three predicted

genes in MAKER2 (see the full annotation on the GitHub page or in the genome browser link provided under Data and materials availability). (**C**) Unique sequences were amplified and their PCR products were visualized in Agarose gel. PCR products were purified from the gel and were Sanger sequenced. (**D**) Alignment of Sanger sequenced amplified products and their sequences in the new assembly (and gel picture) confirm male specificity of these contigs.
